# Western Moyamoya Phenotype: A Scoping Review

**DOI:** 10.7759/cureus.19812

**Published:** 2021-11-22

**Authors:** Raphael Miller, Santiago R Unda, Ryan Holland, David J Altschul

**Affiliations:** 1 Neurological Surgery, Montefiore/Albert Einstein College of Medicine, Bronx, USA

**Keywords:** systematic review, moyamoya disease (mmd), moyamoya angiopathy, article review, clinical phenotype

## Abstract

Moyamoya, a rare angiographic finding, is characterized by chronic and progressive stenosis at the terminal end of the internal carotid artery, followed by collateralization of the cerebral vasculature at the base of the skull. Coined by Suzuki and Takaku in 1969, the term “moyamoya” means a “puff of smoke” in Japanese, a reference to the angiographic appearance of moyamoya collateralization. Moyamoya is most commonly found in East Asian countries, where much governmental and civilian effort has been expended to characterize this unique disease process. However, despite its rarity, the occurrence of moyamoya in Western countries is associated with significant divergence regarding incidence, gender, sex, age at diagnosis, clinical presentation, and outcomes. Here, we attempted to review the Western literature on moyamoya presentation using the PubMed database to characterize the Western phenotype of moyamoya. We were guided by the Preferred Reporting Items for Systematic Reviews and Meta-Analyses extension for scoping reviews (PRISMA-ScR). We reviewed papers generated from a search with keywords “moyamoya case report,” those reported from a Western institution, and those reported on a relevant association. Our scoping review demonstrated various clinical associations with moyamoya. Moreover, we summarized the demographic profile and clinical symptomatology, as well as reported disease associations to better elucidate the Western phenotype of moyamoya.

## Introduction and background

Moyamoya, first described as a distinct disease entity in Japan in 1957, is a chronic and progressive stenosis at the supraclinoid, terminal end of the internal carotid arteries and their proximal branches, and the proximal portions of the middle and anterior cerebral arteries, which can have a unilateral or bilateral presentation. Stenosis of cerebral vasculature leads to the collateralization of the lenticulostriate, leptomeningeal, cortical surface, or Dural vasculature at the base of the skull. A characteristic angiographic finding follows, with a distinct vascular blush on angiography, described by Suzuki and Takaku in 1969 as a “puff of smoke” [1] or “moyamoya” in Japanese. Because of its irreversible and progressive course, Suzuki suggested a time-based classification system that reflects the extent of intracranial artery involvement [1], which is still in use today and is referred to, eponymously, as the Suzuki scale [2].

Moyamoya was originally thought to arise spontaneously and not as a result of a congenital malformation. Indeed, Suzuki wrote about reporting “repeatedly that the disease is an acquired one in its origin resulting from a stricture at the carotid fork due to unknown causes [3].” More recently, in 2011, the *ring finger protein 213* (*RNF213*) gene was identified using genome-wide association studies as the first discovered susceptibility gene for moyamoya [4]. While the exact pathogenic role of *RNF213* in moyamoya is unknown, it has been shown to play a role in angiogenesis [5] and endothelial inflammatory processes [6]. Subsequently, the p.R4810K variant of *RNF213* was identified as a common gene variant, present in 90% of Japanese moyamoya patients, 79% of Korean moyamoya patients, and 23% of Chinese moyamoya patients [7,8]. Other variants of lesser prevalence have also been identified, including the R179 and R258 mutations to the *actin alpha 2* (*ACTA2*) gene [9]. However, the unknown etiology of the remainder, as well as the fact that the p.R4810K *RNF213* variant has not been found in Caucasian moyamoya patients, suggests etiologic heterogeneity for moyamoya vasculopathy [7].

Patients are said to have moyamoya syndrome (MMS) instead of moyamoya disease (MMD) when it occurs together with a well-recognized associated condition, including, but not limited to, sickle cell disease, Down’s syndrome, neurofibromatosis 1, cranial irradiation, type 1 diabetes mellitus, and Graves’ disease [10,11]. MMS is alternatively known as secondary moyamoya or quasi-moyamoya and can be unilateral or bilateral, whereas MMD is alternatively known as primary moyamoya and is, by definition, bilateral [10]. Well-recognized conditions associated with moyamoya are growing in number as research into this strange and mysterious phenotype advances. In this paper, we aim to collate all conditions associated with moyamoya from reports in the Western literature using the PubMed database to elucidate the full spectrum and associations of the Western moyamoya phenotype. Therefore, we chose to avoid the use of these diagnostic descriptors altogether (i.e., primary, secondary, etc.) as the distinction between them is expected to blur as the category of “well-recognized associated conditions” expands. As a secondary goal, we planned to apply similar methodologies to collate and elucidate the symptoms normally associated with moyamoya for greater appreciation of this wide-ranging condition.

MMD and MMS occur mostly in East Asian populations where much governmental and civilian effort has been expended to characterize this unique disease process, which has highlighted important features of the moyamoya phenotype in a broad and robust collection of literature. However, the rarity with which MMS afflicts the Western world presents a unique challenge in the study of its Western phenotype, known to present as a distinct clinical entity [12]. Moreover, various studies in the United States have found rates of MMD increasing over time [13,14], making characterization of associated conditions especially critical. Table 1 references several national in-patient studies reported in the last several decades that are representative of roughly 20% of all US hospital admissions for those years; it demonstrates rising moyamoya rates.

**Table 1:** Relative moyamoya count and male to female ratios in four separate studies. NIS: National Inpatient Sample; NRD: Nationwide Readmissions Database

Study	Reference	Male	Female	N	Inclusion criteria
Moyamoya and inflammation	[15]	30%	70%	2,633*	2009–2012
Prevalence and characteristics of concurrent Down syndrome in patients with moyamoya disease	[16]	29.5%	70.5%	13,275	2002–2009 NIS study of moyamoya patients with Down Syndrome
Moyamoya disorder in the United States	[12]	28%	72%	11,163	2002–2008 NIS study of moyamoya patients
Epidemiological and clinical features of patients with moyamoya disease in the USA)	[14]	29.5%	70.5%	7,473	2005–2008 NIS study of moyamoya patients
Characterization of inpatient moyamoya	[17]	38.1%	61.9%	2,247*	1988–2004 NIS study of moyamoya patients
Treatment course and outcomes after revascularization surgery for moyamoya disease in adults	[13]	25.4%	74.6%	201	2013 NRD study of moyamoya patients that underwent revascularization surgery
Socioeconomic and demographic disparities of moyamoya disease in the United States	[18]	31.7%	68.3%	Not available	2008–2015 NIS study of moyamoya patients

To our knowledge, this is the first attempt to date to collect all known case reports in the literature of Western-type moyamoya for a comprehensive analysis of the unique features with which Western moyamoya can be encountered. Guided by the Preferred Reporting Items for Systematic Reviews and Meta-Analyses extension for scoping reviews (PRISMA-ScR) (Appendices, Figures 2, 3), we aim to answer the research question: what is known about the demographic profiles and clinical symptomatology of Western moyamoya and the diseases that are associated with it?

## Review

Methodology

Protocol and Registration

Our scoping review protocol was not registered.

Eligibility Criteria

We included English-language papers identified by their titles as etiology of moyamoya or moyamoya symptoms. We excluded papers for which the first author’s address was located in Asia (if the first author’s address was unavailable, then the first available address of any author thereafter was used).

Information Sources and Search

A literature search of PubMed was performed to collate case reports on moyamoya. Duplicates were removed manually by the authors. The following search term was used to screen for relevant papers on PubMed published in the preceding 10 years on August 9, 2020: “moyamoya case report.”

Selection of Sources of Evidence

Papers were initially screened by their titles only. However, to better define their status according to the inclusion criteria, the abstract and/or paper were read to clarify ambiguities. Further, for the purpose of the review, relevant papers were read and mined for relevant content. Finally, the association between moyamoya and each symptom and associated pathology was explored in relevant additional literature.

Data Charting Process and Data Items

Data were manually extracted as profiles of patients with major findings relevant to the review and were added to an excel spreadsheet. Unique findings were further reviewed outside of the search criteria for the Discussion section.

Synthesis of Results

Results were grouped based on the association reported by the paper. Additionally, they were classified by the country of the first author’s address according to the inclusion/exclusion criteria.

Results

**Figure 1:**
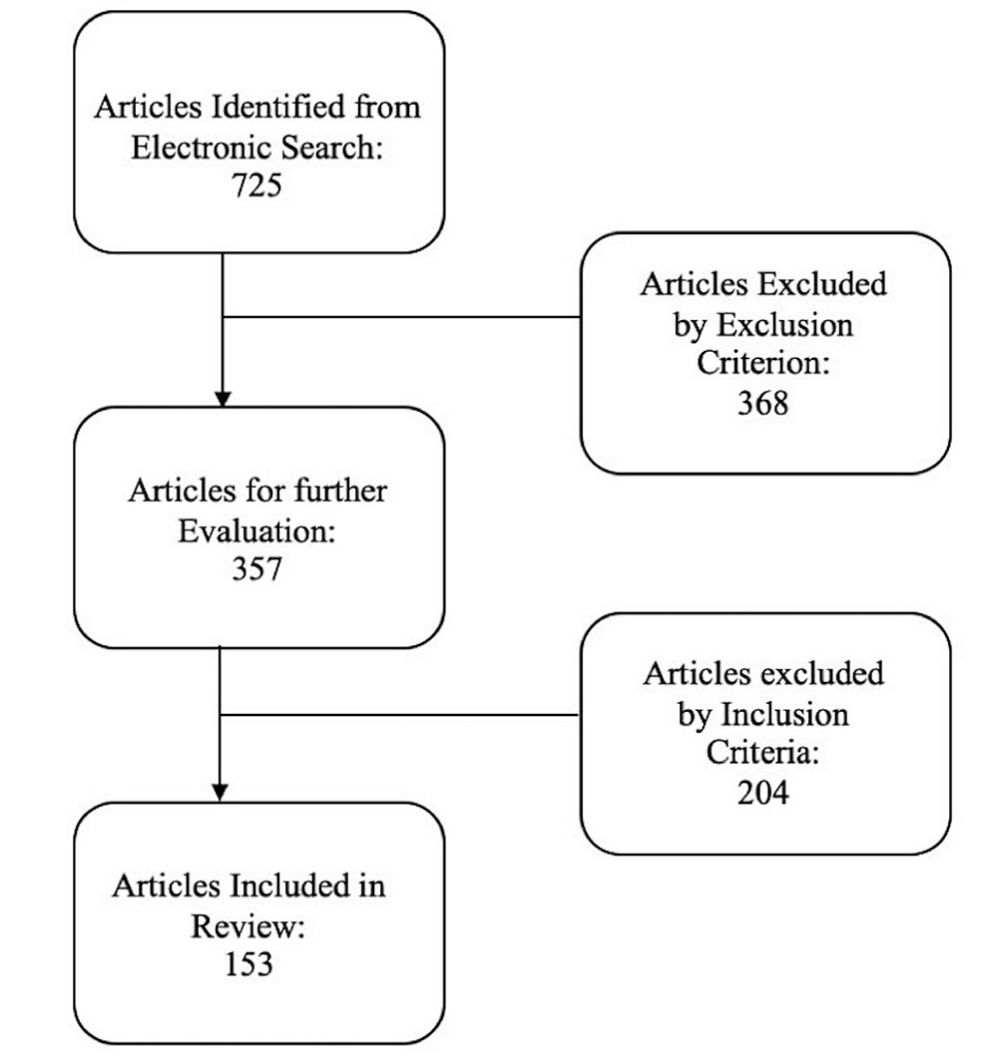
A flowchart schematic of papers identified by the search criteria.

**Figure 2:**
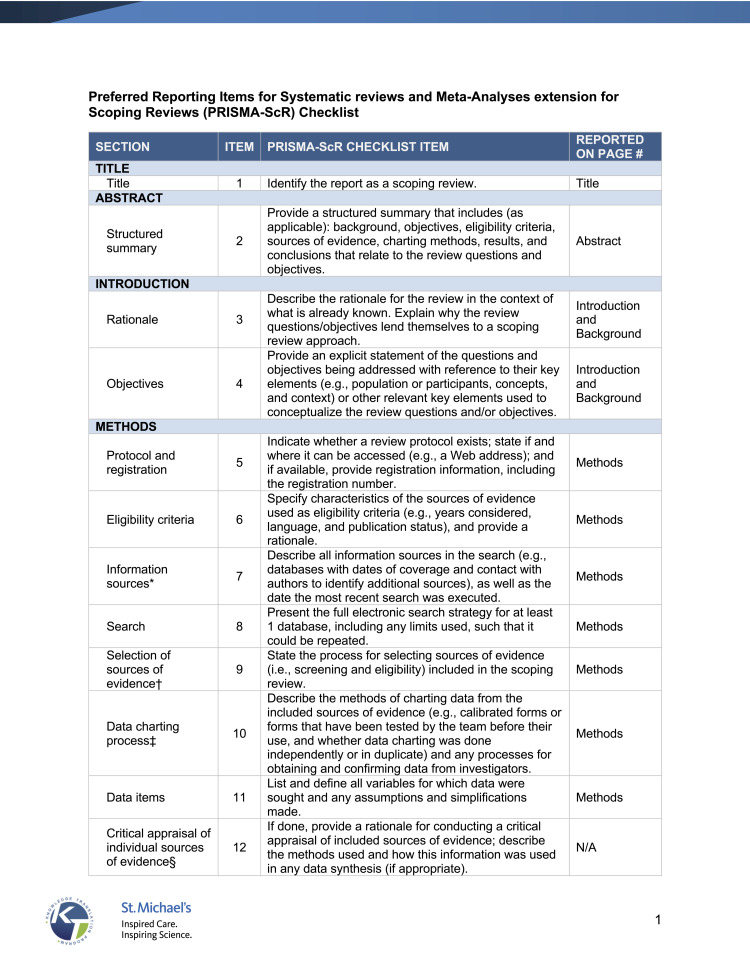
Preferred Reporting Items for Systematic Reviews and Meta-Analyses extension for scoping reviews (PRISMA-ScR) page 1.

**Figure 3:**
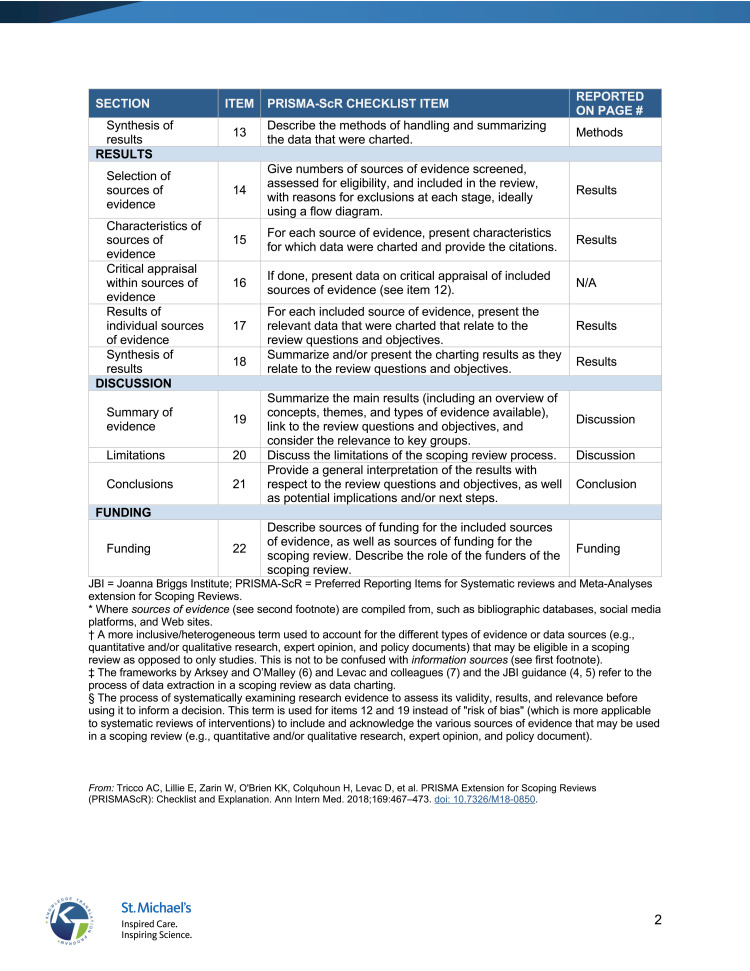
Preferred Reporting Items for Systematic Reviews and Meta-Analyses extension for scoping reviews (PRISMA-ScR) page 2.

Primary search on PubMed using keywords “moyamoya case report” for primary sources, filtered to only include papers published in the last 10 years, yielded 725 papers. A total of 368 papers were removed on applying exclusion criteria, leaving 357 papers. From these papers, 153 were selected for further analysis using our inclusion criteria. See Figure 1 for a flowchart schematic of literature search results.

Table 2 presents a full list of all papers identified by our search criteria.

**Table 2:** List of all papers identified by search criteria, with reference number, PMID, title, and disease/symptom category. *CBL*: *Casitas B-lineage lymphoma*; *RNF213*: *ring finger protein 213*; *KRIT1*: *Krev interaction trapped 1*; *CCM1*: *cavernous malformations-1*; *GUCY1A3*: *guanylate cyclase soluble subunit alpha-3*; PHACE: posterior fossa anomalies, hemangioma, arterial anomalies, cardiac anomalies, and eye anomalies; FIRES: febrile infection-related epilepsy syndrome

Number	Reference	PMID	Title	Category	Association
1	[19]	26795912	Transient intraoperative central diabetes insipidus in moyamoya patients undergoing revascularization surgery: a mere coincidence?	Renal	Diabetes insipidus
2	[20]	23537685	What lies beneath: Fabry nephropathy in a female patient with severe cerebrovascular disease	Renal	Fabry nephropathy
3	[21]	29351941	Moyamoya tipping point: fatal bilateral MCA territory infarction following cocaine abuse	Drugs	Cocaine abuse
4	[22]	20823033	Association of rapidly progressive moyamoya syndrome with bevacizumab treatment for glioblastoma in a child with neurofibromatosis type 1	Drugs	Bevacizumab for glioblastoma
5	[23]	30861282	Tyrosine kinase inhibitor toxicity manifesting as comorbid Moyamoya syndrome and obstructive coronary artery disease: a case report and review of the literature	Drugs	Tyrosine kinase inhibitor toxicity
6	[24]	31658349	Neurofibromatosis type 1 associated with moyamoya syndrome. Case report and review of the literature	Neurocutaneous	Neurofibromatosis type 1
7	[25]	24085541	Moyamoya syndrome related to neurofibromatosis of type 1: a case report	Neurocutaneous	Neurofibromatosis type 1
8	[26]	28279525	“Ivy sign” and moyamoya disease in a child with neurofibromatosis type 1	Neurocutaneous	Neurofibromatosis type 1
9	[27]	24952383	Moyamoya syndrome and neurofibromatosis type 1	Neurocutaneous	Neurofibromatosis type 1
10	[28]	28620564	Moyamoya syndrome in a child with neurofibromatosis type 1: magnetic resonance imaging as a tool for clinical decision making	Neurocutaneous	Neurofibromatosis type 1
11	[29]	29364453	Moyamoya syndrome associated with neurofibromatosis type 1 in a pediatric patient	Neurocutaneous	Neurofibromatosis type 1
12	[30]	24801636	Adult-onset moyamoya disease in a patient with neurofibromatosis type 1	Neurocutaneous	Neurofibromatosis type 1
13	[31]	23661665	Simultaneous moyamoya disease and cervical spinal cord low-grade astrocytoma in a child with neurofibromatosis type 1	Neurocutaneous	Neurofibromatosis type 1
14	[32]	21603789	Moyamoya syndrome associated with neurofibromatosis type I in a pediatric patient	Neurocutaneous	Neurofibromatosis type 1
15	[33]	21271658	Cerebrovasculopathy in NF1 associated with ocular and scalp defects	Neurocutaneous	Neurofibromatosis type 1
16	[34]	24141273	Poor wound healing after pial synangiosis in 2 children with moyamoya vasculopathy associated with neurofibromatosis type 1	Neurocutaneous	Neurofibromatosis type 1
17	[35]	28237043	Tuberous sclerosis complex and polycystic kidney disease contiguous gene syndrome with moyamoya disease	Neurocutaneous	Tuberous sclerosis complex
18	[36]	24878482	Moyamoya syndrome associated with mucolipidosis-II	Metabolic	Mucolipidosis 2
19	[37]	25207193	Protein S deficiency and an adult case with moyamoya syndrome that presented with primary intraventricular haemorrhage	Metabolic	Protein S deficiency
20	[38]	29843966	CD59 deficiency presenting as polyneuropathy and moyamoya syndrome with endothelial abnormalities of small brain vessels	Metabolic	CD59 deficiency
21	[39]	30021915	Severe hyperhomocysteinemia manifesting as moyamoya vasculopathy and Henoch-Schonlein purpura	Metabolic	Hyperhomocysteinuria
22	[40]	27321952	Unusual association between lysinuric protein intolerance and moyamoya vasculopathy	Metabolic	Lysinuric protein intolerance
23	[41]	22034792	Moyamoya syndrome with arteriovenous dural fistula after head trauma	Physical trauma	Head trauma
24	[42]	21830458	Moyamoya disease in a patient with brain tumor: case report	Physical trauma	Brain tumor
25	[43]	28698088	Microsurgical repair of ruptured aneurysms associated with moyamoya-pattern collateral vessels of the middle cerebral artery: a report of two cases	Physical trauma	Postsurgical repair of a ruptured aneurysm
26	[44]	27857839	Fatal outcome in a Hispanic woman with moyamoya syndrome and Graves’ disease	Autoimmune	Graves’ disease
27	[45]	21863249	Dual anca positivity in a child with moyamoya-like cerebral vascular changes: an unusual presentation with sudden homonymous hemianopsia	Autoimmune	Dual antineutrophil cytoplasmic antibodies positivity
28	[46]	30017593	Moyamoya vasculopathy with anti-SCL-70 antibodies: a case report and review of the literature	Autoimmune	Anti-SCL-70 antibodies
29	[47]	30732990	Reply to ‘Moyamoya vasculopathy with anti-SCL-70 antibodies: A case report and review of the literature’	Autoimmune	Anti-SCL-70 antibodies
30	[48]	23651859	Systemic lupus erythematosus due to C1q deficiency with progressive encephalopathy, intracranial calcification and acquired moyamoya cerebral vasculopathy	Autoimmune	SLE secondary to C1q deficiency
31	[49]	30094527	Moyamoya complicated by thrombotic cerebrovascular accident in a Caucasian woman with collagenous colitis	Autoimmune	Collagenous colitis
32	[50]	27606722	Moyamoya disease and systemic sclerosis (MoSys syndrome): a combination of two rare entities	Autoimmune	Systemic sclerosis
33	[51]	23380559	Concurrent Takayasu arteritis with common variable immunodeficiency and moyamoya disease	Autoimmune	Takayasu arteritis
34	[52]	27592246	Moyamoya syndrome causing stroke in young women with type 1 diabetes	Autoimmune	Diabetes mellitus type 1
35	[53]	21130668	Moyamoya syndrome associated with Graves’ disease: a case report and review of the literature	Autoimmune	Graves’ disease
36	[54]	29475609	Moya-moya syndrome after cranial radiation for optic glioma with NF1. Case report and literature review of syndromic cases	Radiation	Cranial radiation for optic glioma
37	[55]	27193755	Radiation-induced moyamoya syndrome after proton beam therapy in the pediatric patient: a case series	Radiation	Proton beam therapy
38	[56]	21783367	Radiation-induced moyamoya disease after childhood astrocytoma	Radiation	Radiation for childhood astrocytoma
39	[57]	24501091	The development of moyamoya syndrome after proton beam therapy	Radiation	Proton beam therapy
40	[58]	30583132	Radiation-induced moyamoya syndrome after proton therapy in child with clival chordoma: natural history and surgical treatment	Radiation	Proton beam therapy for clival chordoma
41	[59]	31893204	Moyamoya syndrome after radiation therapy: a clinical report	Radiation	Radiation therapy
42	[60]	28343148	De novo mutations in CBL causing early-onset paediatric moyamoya angiopathy	Congenital	CBL mutations
43	[61]	26277359	Familial moyamoya disease in two Turkish siblings with same polymorphism in RNF213 gene but different clinical features	Congenital	RNF213 polymorphism
44	[62]	30463371	Microduplication of 15q13.3 and microdeletion of 18q21.32 in a patient with moyamoya syndrome	Congenital	Microduplication of 15q13.3 and microdeletion of 18q21.32
45	[63]	29263223	Moyamoya-like cerebrovascular disease in a child with a novel mutation in myosin heavy chain 11	Congenital	Myosin heavy chain 11 mutation
46	[64]	27236536	Moyamoya syndrome and 6p chromosome rearrangements: expanding evidences of a new association	Congenital	6p chromosome rearrangements
47	[65]	25413039	Cerebral cavernous malformations and unilateral moyamoya in a patient with a new mutation in the KRIT-1/CCM1 gene	Congenital	KRIT1/CCM1 mutation
48	[66]	26777256	Disrupted nitric oxide signaling due to GUCY1A3 mutations increases risk for moyamoya disease, achalasia and hypertension	Congenital	GUCY1A3 mutation
49	[67]	28686325	RNF213 variants in a child with PHACE syndrome and moyamoya vasculopathy	Congenital	RNF213 variant, PHACE syndrome
50	[68]	26198278	Atypical presentation of moyamoya disease in an infant with a de novo RNF213 variant	Congenital	RNF213 variant
51	[69]	27611897	Surgical outcomes of Majewski osteodysplastic primordial dwarfism type II with intracranial vascular anomalies	Congenital	Majewski osteodysplastic primordial dwarfism type II
52	[70]	22527565	Medical management of moyamoya disease and recurrent stroke in an infant with Majewski osteodysplastic primordial dwarfism type II (MOPD II)	Congenital	Majewski osteodysplastic primordial dwarfism type II
53	[71]	23337351	“Ocular moyamoya” syndrome in a patient with features of microcephalic osteodysplastic primordial dwarfism type II	Congenital	Majewski osteodysplastic primordial dwarfism type II
54	[72]	28926972	Juvenile moyamoya and craniosynostosis in a child with deletion 1p32p31: expanding the clinical spectrum of 1p32p31 deletion syndrome and a review of the literature	Congenital	1p32p31 deletion
55	[73]	21147392	Moyamoya disease associated with hereditary spherocytosis	Hematological	Hereditary spherocytosis
56	[74]	28612582	Management in rare association of moyamoya syndrome and hereditary spherocytosis	Hematological	Hereditary spherocytosis
57	[75]	28221268	Moyamoya syndrome associated with hereditary spherocytosis: an emerging clinical entity	Hematological	Hereditary spherocytosis
58	[76]	22606383	Laparoscopic splenectomy in a child with moyamoya syndrome, hereditary spherocytosis, and interstitial lung disease: a mere coincidence or partnership based on genetic similarities	Hematological	Hereditary spherocytosis
59	[77]	21728723	Cerebral infarction in the setting of moyamoya in a pediatric patient with sickle β+-thalassemia	Hematological	Sickle β+-thalassemia
60	[78]	25719591	Fanconi anemia associated with moyamoya disease in Saudi Arabia	Hematological	Fanconi anemia
61	[79]	25178886	Moyamoya syndrome in sickle cell anaemia: a cause of recurrent stroke	Hematological	Sickle cell anemia
62	[80]	24948625	Novel severe hemophilia A and moyamoya (SHAM) syndrome caused by Xq28 deletions encompassing F8 and BRCC3 genes	Hematological	Hemophilia A
63	[81]	25342087	Moyamoya syndrome associated with hemoglobin Southampton (Casper)	Hematological	Hemoglobin Southampton
64	[82]	23735661	A rare case of moyamoya syndrome in a β-thalassemia major patient	Hematological	β-thalassemia major patient
65	[83]	31100171	CRAO in moyamoya syndrome associated with Southampton hemoglobinopathy	Hematological	Hemoglobin Southampton
66	[84]	26422091	X-linked moyamoya syndrome associated with severe haemophilia A	Hematological	Hemophilia A
67	[85]	23953970	Moyamoya syndrome associated with sickle cell trait in a child	Hematological	Sickle cell trait
68	[86]	23369159	Moyamoya syndrome with sickle cell trait	Hematological	Sickle cell trait
69	[87]	24571831	Ivy sign in mildly symptomatic β-thalassemia intermedia, with development of moyamoya disease	Hematological	β-thalassemia intermedia
70	[88]	27843469	Moyamoya syndrome associated with Henoch-Schönlein purpura	Hematological	Henoch-Schönlein purpura
71	[89]	21337683	Oculoectodermal syndrome with coarctation of the aorta and moyamoya disease: expanding the phenotype to include vascular anomalies	Syndrome	Oculoectodermal syndrome
72	[90]	28647711	Down syndrome and moyamoya disease: unusual cause of stroke	Syndrome	Down syndrome
73	[91]	26778511	Management of moyamoya syndrome in patients with Noonan syndrome	Syndrome	Noonan syndrome
74	[92]	24397103	Childhood moyamoya disease accompanying Leigh syndrome	Syndrome	Leigh syndrome
75	[93]	22759690	Moyamoya vascular pattern in Alagille syndrome	Syndrome	Alagille syndrome
76	[94]	24103673	Moyamoya disease associated with asymptomatic mosaic Turner syndrome: a rare cause of hemorrhagic stroke	Syndrome	Turner syndrome
77	[95]	25307898	Moyamoya syndrome or Behçet's disease?	Syndrome	Behcet’s disease
78	[96]	25528372	Moyamoya in a patient with Sneddon's syndrome	Syndrome	Sneddon’s syndrome
79	[97]	30761079	Extracranial and intracranial vasculopathy with “moyamoya phenomenon” in association with Alagille syndrome	Syndrome	Alagille syndrome
80	[98]	28380489	Moyamoya in a patient with Smith-Magenis syndrome	Syndrome	Smith-Magenis syndrome
81	[99]	26043700	Pretransplant management of basilar artery aneurysm and moyamoya disease in a child with Alagille syndrome	Syndrome	Alagille syndrome
82	[100]	23692605	Moyamoya syndrome in a Malaysian child with Down syndrome	Syndrome	Down syndrome
83	[101]	28436815	Moyamoya in a patient with FIRES: a first case report	Syndrome	FIRES
84	[102]	26576049	Anesthetic management of a parturient with PHACE syndrome for cesarean delivery	Syndrome	PHACE syndrome
85	[103]	26844876	Moyamoya disease in a patient with VACTERL association	Syndrome	VACTERL association
86	[104]	26540473	Morning glory syndrome with carotid and middle cerebral artery vasculopathy	Syndrome	Morning glory syndrome
87	[105]	28859027	Subarachnoid hemorrhage revealing moyamoya syndrome in a patient with May-Hegglin anomaly	Syndrome	May-Hegglin anomaly
88	[106]	22541517	Association of mesial temporal sclerosis and moyamoya syndrome	Syndromes	Mesial temporal sclerosis
89	[107]	23560742	Moyamoya disease with mesial temporal sclerosis	Syndrome	Mesial temporal sclerosis
90	[108]	24172590	Presentation with recurrent intractable headache: a patient with moyamoya syndrome--case report	Headache	Headache
91	[109]	31245213	A case report of moyamoya disease presenting as headache in a 35-year-old Hispanic man	Headache	Headache
92	[110]	29902294	Bilateral visual field loss in an adolescent girl with migraine headaches	Headache	Migraine
93	[111]	22425396	Migraine-like headache and ocular malformations may herald moyamoya syndrome	Headache	Migraine
94	[112]	28132315	O063. Moyamoya disease and headache: case report	Headache	headache
95	[113]	23391943	Postpneumococcal moyamoya syndrome case report and review of the postinfective cases	Post-infection	Postpneumococcal
96	[114]	32434020	A rare case of postinfectious moyamoya syndrome: case report and review of the literature	Post-infection	Postinfectious
97	[115]	28411258	Patient with severe moyamoya disease who presents with acute cortical blindness	Ophthalmologic	Acute cortical blindness
98	[116]	25799075	Ocular ischemic syndrome presenting as retinal vasculitis in a patient with moyamoya syndrome	Ophthalmologic	Ocular ischemic syndrome
99	[117]	24515702	“The fainted man:” hypoperfusion encephalopathy in a patient with moyamoya	Neurological	Hypoperfusion encephalopathy
100	[118]	30554704	Neurologically acquired laryngomalacia in a pediatric patient with moyamoya: A case report and literature review	Neurological	Laryngomalacia
101	[119]	30564539	Depression and catatonia: a case of neuropsychiatric complications of moyamoya disease	Neurological	Neuropsychiatric complications
102	[120]	31868692	Neuropsychological profile associated with moyamoya disease: A case report	Neurological	Neuropsychological profile
103	[121]	30929500	Psychiatric comorbidity in moyamoya disease and preliminary guidelines for treatment	Neurological	Psychiatric comorbidity
104	[122]	29179236	Moyamoya syndrome manifesting with choreiform movements	Neurological	Choreiform movements
105	{Platzen, 2017 #1419}[123]	28526173	Chorea gravidarum associated with moyamoya angiopathy treated with alpha-methyldopa	Neurological	Chorea gravidarum
106	{Laatar, 2017 #1145}[124]	29878297	Generalized dystonia : unusual mode of revelation of moyamoya disease	Neurological	Generalized dystonia
107	[125]	26450280	Pial synangiosis ameliorates movement disorders in the absence of prior stroke in moyamoya disease	Neurological	Movement disorders
108	[126]	24022555	An unusual cause of hemiplegia in a 28-year-old woman	Neurological	Hemiplegia
109	[127]	21121731	Patients with moyamoya disease presenting with movement disorder	Neurological	Movement disorder
110	[128]	27849243	Moyamoya disease: impact on the performance of oral and written language	Neurological	Oral and written language deficits
111	{Zalonis, 2010 #2694}[129]	21323131	Cognitive deficits presenting as psychiatric symptoms in a patient with moyamoya disease	Neurological	Cognitive deficits
112	[130]	23793182	Hemifacial spasm leading to diagnosis of moyamoya disease	Neurological	Hemifacial spasm
113	[131]	28698731	Intermittent hemiplegia in a boy with primary moyamoya disease: a case report from Iran	Neurological	Intermittent hemiplegia
114	[132]	26890714	Unilateral sudden hearing loss: a rare symptom of moyamoya disease	Neurological	Hearing loss
115	[133]	25982655	Transcatheter creation of a reverse Potts shunt in a patient with severe pulmonary arterial hypertension associated with moyamoya syndrome	Vascular	Pulmonary arterial hypertension
116	[134]	29724978	Atypical presentation of moyamoya disease with pulmonary hypertension: a case report	Vascular	Pulmonary hypertension
117	[135]	28946753	6-year-old with severe hypertension	Vascular	Hypertension
118	[136]	23430230	Ophthalmic artery ischemic syndrome associated with neurofibromatosis and moyamoya syndrome	Vascular	Ophthalmic artery ischemic syndrome
119	[137]	20526643	Moyamoya disease and arteriovenous fistula of the epiaortic vessels	Vascular	Arteriovenous fistula of the epiaortic vessels
120	[138]	32705354	Central retinal artery occlusion occurring 30 years after successful revascularization surgery for moyamoya disease: case report	Vascular	Central retinal artery occlusion
121	[139]	26605691	Retinal arterial tortuosity in moyamoya disease	Vascular	Retinal arterial tortuosity
122	[140]	27928391	A moyamoya patient with bilateral consecutive branch retinal vein occlusion	Vascular	Retinal vein occlusion
123	[141]	23394336	Intracranial atherosclerotic disease associated with moyamoya collateral formation: histopathological findings	Vascular	Intracranial atherosclerotic disease
124	[142]	25323944	Hemodynamic features of non-aneurysmal subarachnoid hemorrhage in a case of familial moyamoya disease: a transcranial Doppler ultrasound study	Vascular	Non-aneurysmal subarachnoid hemorrhage
125	[143]	29367356	Multiple anomalies in the origin and course of vertebral arteries and aberrant right subclavian artery in a child with moyamoya syndrome	Vascular	Anomalies in the origin and course of vertebral arteries and aberrant right subclavian artery
126	[144]	28808729	Twig-like middle cerebral artery: a seldom vessel anomaly of important relevance	Vascular	Twig-like middle cerebral artery
127	[145]	20809708	Diffuse and uncontrolled vascular smooth muscle cell proliferation in rapidly progressing pediatric moyamoya disease	Vascular	Vascular smooth muscle cell proliferation
128	[146]	25773096	Central retinal vein occlusion with bilateral stenosis of the internal carotid arteries	Vascular	Central retinal vein occlusion
129	[147]	26864187	Peripheral pulmonary artery stenoses in the setting of moyamoya	Vascular	Peripheral pulmonary artery stenoses
130	[148]	32322754	Central retinal artery occlusion as initial presentation of moyamoya disease in a middle-aged woman	Vascular	Central retinal artery occlusion
131	[149]	30978680	Development of bilateral dural arteriovenous fistulae following pial synangiosis for moyamoya syndrome: case report	Vascular	Bilateral dural arteriovenous fistulae
132	[150]	30863645	ST-elevation myocardial infarction (STEMI) in a patient with moyamoya disease	Vascular	ST-elevation myocardial infarction
133	[151]	22742664	Differential diagnosis between cerebral ischemia, focal seizures and limb shaking TIAs in moyamoya disease	Stroke	Seizures, transient ischemic attack, cerebral ischemia
134	[152]	32733722	Postpartum-onset moyamoya disease: a rare cause of stroke in unexpected	Stroke	Postpartum stroke
135	[153]	31885602	Subacute stroke in a young female: a case of moyamoya syndrome initially anchoring with anxiety	Stroke	Subacute stroke
136	[154]	30538867	Bilateral cerebrovascular stroke as an initial presenting symptom of moyamoya disease	Stroke	Bilateral cerebrovascular stroke
137	[155]	32010532	A case of delayed diagnosis of moyamoya disease after recurrent strokes	Stroke	Stroke
138	[156]	30093460	Peek through the smoke: a report of moyamoya disease in a 32-year-old female patient presenting with ischaemic stroke	Stroke	Ischemic stroke
139	[157]	22547520	Multiple cerebral infarcts in patient with moyamoya disease	Stroke	Stroke
140	[158]	27252954	Occult moyamoya disease causing fulminant infarction after septorhinoplasty	Stroke	Stroke
141	[159]	25496692	Endovascular treatment of a ruptured flow aneurysm of the heubner artery as part of a moyamoya collateral network in a young patient with an occluded middle cerebral artery	Aneurysm	Fulminant stroke
142	[160]	23169511	Moyamoya disease misdiagnosed as leptomeningeal metastases	Aneurysm	Leptomeningeal metastases
143	[161]	27236734	Ruptured posterior ethmoidal artery aneurysm and moyamoya disease in an adult patient. Case report	Aneurysm	Ruptured posterior ethmoidal artery aneurysm
144	[162]	20075103	Embolization of an unruptured distal lenticulostriate aneurysm associated with moyamoya disease	Aneurysm	Unruptured distal lenticulostriate aneurysm
145	[163]	29468103	Atypical location of intracerebral hemorrhage in moyamoya disease	Hemorrhage	Intracerebral hemorrhage
146	[164]	21748033	Non-aneurysmal subarachnoid hemorrhage as presentation of moyamoya disease in an adult	Hemorrhage	Non-aneurysmal subarachnoid hemorrhage
147	[165]	31689569	Pediatric moyamoya presenting as a subarachnoid hemorrhage from a ruptured anterior cerebral artery aneurysm	Hemorrhage	Subarachnoid hemorrhage from a ruptured anterior cerebral artery aneurysm
148	[166]	20198515	Transcranial Doppler ultrasound CO_2_ challenge complicated by subarachnoid hemorrhage in patient with moyamoya syndrome	Hemorrhage	Subarachnoid hemorrhage
149	[167]	31934477	Postpartum seizure and subarachnoid haemorrhage secondary to moyamoya disease	Hemorrhage	Subarachnoid hemorrhage
150	[168]	28215908	Intraparenchymal haemorrhage secondary to moyamoya disease in a white patient	Hemorrhage	Intraparenchymal hemorrhage
151	[169]	23549955	Fatal hemorrhagic stroke in a Caucasian girl with moyamoya disease	Hemorrhage	Fatal hemorrhagic stroke
152	[170]	29482684	Dental management of a pediatric patient with moyamoya syndrome: a rare clinical entity	Dental	Dental caries, stroke risk
153	[171]	28814371	Dental management considerations in a pediatric patient with moyamoya disease	Dental	Dental care, stroke risk

We categorized the 153 papers into two categories that will be discussed separately. Overall, 44 papers reported a symptom associated with moyamoya, and 109 reported a condition associated with moyamoya.

Symptomatology

Table 3 tabulates symptoms reported in 44 papers describing a moyamoya symptom association.

**Table 3:** List of symptom categories associated with moyamoya.

Symptom category	N (%)
Aneurysm	4 (9)
Dental	2 (5)
Headache	5 (11)
Hemorrhage	7 (16)
Neurologic	16 (36)
Ophthalmologic	2 (5)
Stroke	8 (18)
Total	44

None of the previously mentioned large database studies reported aneurysms as a primary reported endpoint, possibly because of its rarity in Western patients. One study in Germany published in 2016 of 55 moyamoya patients showed that 37 (67.3%) had suffered from a headache in the last year, with 47.9% having migraine-like headaches, of which 58.8% of the cases described migraine aura [109,172]. The rates of hemorrhage reported in several large studies are presented in Table 4.

**Table 4:** Rates of hemorrhage in several large studies of moyamoya patients.

Study name	Reference	Study duration	N	Findings
Characterization of inpatient moyamoya	[17]	1988–2004	2,247	11.1% hemorrhagic events
Epidemiological and clinical features of patients with moyamoya disease in the USA	[14]	2005–2008	7,473	6.1% hemorrhagic strokes
Treatment course and outcomes after revascularization surgery for moyamoya disease in adults	[13]	2013	201	3.5% hemorrhagic events

In individual moyamoya patients, neurological symptoms secondary to moyamoya included laryngomalacia [118], hypotension encephalopathy [117], behavioral changes [119], oral and written language deficits [119,128], transient psychosis [121,129,173], hemiplegia [126,131], hearing loss [132], and cognitive difficulties [120]. Movement disorders as symptoms of moyamoya included chorea [122,123], dystonia [124,125], hemifacial spasms [130], and hemiballismus [127]. Moreover, Kraemer et al. analyzed the incidence of movement disorders among moyamoya patients in Europe. Using self-rating questionnaires submitted by 59 patients (13 males, 36 females), more than half (54.9%) reported a history of movement disorders. Additionally, in response to a multiple-choice question requesting descriptive data on movement disorders, 7% chose periodic tremors, 33.3% irregular jerks, 20% involuntary stiffness and cramps, and 15.7% unintended movement with loopy or pranced character [174]. Another study examined the clinical records of 143 moyamoya patients (89 females, 54 males), of which 16 were Asian, and reported only six cases of choreiform movement disorders (4.2%) [175]. This wide discrepancy is likely the result of diverging classification schemes.

A case of central deafness in a Caucasian child with moyamoya was reported. The child was a three-year-old male with no known Asian ancestry and was presumed to have suffered developmental retardation because of his cerebrovascular stenosis [176].

Regarding ophthalmologic symptoms, studies have been reported of a moyamoya patient with acute cortical blindness [115] and another with ocular ischemic syndrome [116].

The most commonly reported stroke type was ischemic infarction. In National Inpatient Sample (NIS) studies of disease profiles of moyamoya patients in the United States, ischemic stroke was the most common co-occurring symptom, with an incidence of 20.7% in the 1988-2004 study and 11.6% in the 2005-2008 study. In the 2013 Nationwide Readmission Database (NRD) study, 5% of moyamoya patients presenting for revascularization surgery were coded for ischemic stroke.

In the United States, symptomatic moyamoya patients have lower rates of hemorrhagic stroke than their Japanese and Korean counterparts (33.6% vs. 61% and 59%). This supports the notion that moyamoya outside of the West is pathophysiologically distinct from its presentation in East Asia. Additionally, hemorrhagic stroke was more frequent in adults than in children (18.1% vs. 1.5%, p < 0.05) [14]. Further, 20.7% of patients in the NIS study from 1988 to 2004 were diagnosed with ischemic stroke, 7.4% with intracerebral hemorrhage, 3.1% subarachnoid hemorrhage, 3.4% transient ischemic attack (TIA), 13.5% anemia, and 13.6% with sickle cell disease or trait [17]. A higher percentage of moyamoya patients in the West have an identifiable underlying condition than their East Asian counterparts [177].

Additionally, the 2018 study of moyamoya patients who underwent revascularization surgery found that vascular risk factors were common, with one out of four having diabetes, half having hypertension, and 40% each having hypocholesteremia or a history of smoking. Further, moyamoya has also been reported in patients with fibromuscular dysplasia [178]. Renovascular hypertension has also been reported in association with moyamoya [179].

Disease Associations

Table 5 lists the disease categories associated with moyamoya.

**Table 5:** Disease categories associated with moyamoya.

Disease category	N (%)
Autoimmune	10 (9)
Congenital	13 (12)
Drugs	3 (3)
Hematologic	16 (15)
Metabolic	5 (5)
Neurocutaneous	12 (11)
Physical trauma	3 (3)
Postinfectious	2 (2)
Radiation therapy	6 (6)
Renal	2 (2)
Syndromes	19 (17)
Vascular	18 (17)
Total	109

We found 10 (9%) papers describing an association between moyamoya and an autoimmune condition. This finding is consistent with previous reports of associations between MMS and autoimmune diseases in Eastern cohorts [180,181]. In 2012, one research group at the Mayo Clinic in Rochester reported an unusually high prevalence of autoimmune diseases among their cohort of mostly white, mid-Western patients. Later, in 2016, another group in Washington reported four cases of MMS with pre-existing type 1 diabetes mellitus. Two of the patients also had Graves’ disease, and another had systemic lupus erythematosus [52]. Then, in 2017, researchers analyzed 2,633 patients in the NIS dataset from 2009 to 2012 and reported a strong (p < 0.05) association between adult-onset autoimmune diseases and moyamoya (NIS lacks distinction between MMS and MMD) in the pediatric population (but not the adult population). The adult-onset autoimmune diseases included Addison’s disease, dermatomyositis, granulomatosis with polyangiitis, Graves’ disease, multiple sclerosis, myasthenia gravis, polymyositis, primary systemic vasculitis, rheumatoid arthritis, Sjogren’s disease, systemic lupus erythematosus, systemic sclerosis, and thyroiditis. By contrast, juvenile-onset autoimmune diseases, including diabetes mellitus type 1 and juvenile rheumatoid arthritis, were associated with moyamoya in both the pediatric and adult populations [15]. Finally, in 2018, 26% of 31 mostly Caucasian MMD patients in Kentucky were reported to have a co-existing autoimmune condition, including rheumatoid arthritis, lupus, hypothyroidism, psoriasis, polyglandular autoimmune type 1, autoimmune hepatitis, Addison’s disease, immune thrombocytopenic purpura, Crohn’s disease, multiple sclerosis, celiac sprue, and dermatitis herpetiformis [182]. Other reports have shown an association between moyamoya and febrile infection-related epilepsy syndrome (FIRES) [101], Graves’ disease [182], and collagenous colitis [183]. Of note, not a single case of co-existing moyamoya and Sjogren’s syndrome could be found for a Western patient.

The association between autoimmunity and moyamoya has been thoroughly demonstrated using animal models. Half a century ago, researchers noticed that moyamoya patients were experiencing leptospirosis complications, in which 81.4% of their cerebral spine fluid had a positive immune response. Later, in 1983, Japanese researchers observed that moyamoya patients were at a greater risk for tonsillitis, otitis media, maxillary sinusitis, and fever and infection of unknown origin. However, more recent research has called into question the proposed autoimmune etiology of the moyamoya phenotype [184].

We found several reports showing an association between moyamoya and Majewski osteodysplastic primordial dwarfism type 2 (MOPD2) [69-71,185], a rare autosomal recessive disorder. Two studies demonstrated this as well. One study found 11 cases of moyamoya in the literature out of 58 mostly Western patients (19%) with MOPD2 in 2004 [183], while another found 13 (52%) among 25 patients with MOPD2 [186]. Moyamoya has been reported in Western patients in association with other rare growth disorders, including Seckel syndrome [187,188] Cockayne syndrome [189], floating harbor syndrome [190], and Noonan syndrome [91].

Moyamoya has been reported in association with glycogen storage disease type 1a in France [191] and the United States [192]. Additionally, it has been reported in association with Alagille syndrome [93,193], a rare, autosomal dominant hepatic disorder. A few cases of moyamoya have been reported in patients with Turner’s syndrome [94,193]. In our literature search, we found one case of moyamoya with Aicardi Goutieres syndrome [194].

We found three (3%) papers describing an association between moyamoya and drugs. Oral contraceptives have been recognized as a potential risk factor for moyamoya in the West since at least 1984 [195]. A small cohort study conducted in Canada and the United States consisting of 39 patients with moyamoya [196] and another literature review of moyamoya cases in the United States [183], both in 1997, showed that oral contraceptive use was associated with moyamoya. We found one report of cocaine abuse associated with moyamoya [21]. Another two articles reported an association with nilotinib, a second-generation tyrosine kinase inhibitor [23], and bevacizumab [22].

We found 16 (15%) papers describing an association between moyamoya and hematologic disorders. These include hereditary spherocytosis [124-127], beta-thalassemia [128,133,173], Fanconi anemia [78], sickle cell anemia [79] and trait [85,86], hemophilia A [131,197], hemoglobin Southamptom [132], and Henoch-Schonlein purpura [174].

Sickle cell anemia predisposes patients to internal carotid artery stenosis, leading to moyamoya [198]. A 2019 study identified 61 studies in the literature on sickle cell-associated moyamoya revascularization surgery outcomes, presumably a significant number of which occurred in the Western population [199]. A 2011 NIS study of moyamoya admissions from 1988 to 2004 showed that 13.6% of all moyamoya patients had sickle cell disease or trait at admission [17].

To our knowledge, five cases of MMS with hereditary spherocytosis have been reported [75], of which at least three were Western patients.

Additionally, East Asian researchers have reported at least five cases of moyamoya associated with paroxysmal nocturnal hemoglobinuria [200], although to our knowledge, none have been reported to date in Western patients. Additional hematological conditions not identified by our search criteria included hemoglobin Fairfax-beta-thalassemia in 2008 in Indiana [201] and hemoglobin E/beta-thalassemia in 2009 in Cambodia [202].

We found five papers (5%) that described an association between moyamoya and metabolic disorders, including mucolipidosis II [36], protein S deficiency [37], CD59 deficiency [38], severe hyperhomocysteinemia [39], and lysinuric protein intolerance [40].

We found 12 (11%) papers describing an association between moyamoya and Neurocutaneous disorders, including one case of tuberous sclerosis [35] and the rest of neurofibromatosis type 1 (NF1) [24-34]. One study found approximately 250 children with NF1 in the literature since 1976 [12], and another NIS study found 51 of 2,247 moyamoya patients with NF between 1988 and 2004 [17]. NF1 is an autosomal dominant genetic neurocutaneous disorder caused by a mutation in the *NF1 *gene, located on chromosome 17 (17q11.2). The disorder leads to a large spectrum of central nervous system manifestations, including learning disabilities, mental retardation, seizures, attention deficit with hyperkinesia disorder, neurofibromas, and optic nerve glioma. While the mechanism by which NF1 leads to moyamoya is unknown, it is probably related to the function of the protein encoded by the *NF1 *gene, a negative regulator of Ras. This is especially plausible because moyamoya is known to be associated with RASopathies, including Noonan syndrome and Costello syndrome.

Associations between tumors and moyamoya have been reported in East Asian literature, but they are very rare [203]. Furthermore, we found two (2%) papers that described an association between moyamoya and a post-infection state, including meningitis secondary to *Aspergillus fumigatus* and *Escherichia coli* [114] and pneumococcal meningitis [113].

On further analysis, we found several other reports of post-infectious moyamoya, including quaternary neurosyphilis in 1989 [204] and *Haemophilus influenaze* type C meningitis in Texas in 2003 [205]. Moreover, one national multicenter study identified five South African children (four girls of indigenous African ancestry) with human immunodeficiency virus-associated vasculopathy and MMS [206], and a Canadian and US cohort demonstrated an association between moyamoya and tuberculosis in 1997 [196]. Finally, a patient with acquired immunodeficiency syndrome was reported as presenting concurrently with moyamoya [207].

We found six (6%) papers describing an association between moyamoya and radiation therapy. Radiation therapy is a known risk factor for the development of cerebrovascular pathologies. A recent 2019 PubMed analysis of case reports of radiation-induced moyamoya found 54 reported cases [208]. Proton beam therapy, a safer alternative to traditional radiation therapy, has also been associated with moyamoya [55]. One of the first reported cases of moyamoya associated with radiation therapy in the United States was published in 1978 [209], followed by what was probably the first cohort study of the association in Western patients in Toronto, Ontario in 1993 with five patients treated for optic gliomas [210]. All patients were Caucasian, and four received 5,000 rad of radiation or more and one received 2,500 rad. In four cases, the presentation was ischemic and two patients presented with TIAs. While there have been several recent meta-analyses of the literature regarding the association between radiation therapy and moyamoya in the global population, there have been none published to date specifically for Western populations to our knowledge.

We found 19 (17%) papers that described an association between moyamoya and rare syndromes, including oculoectodermal syndrome [89], Down syndrome [90, 100], Noonan syndrome [91], Leigh syndrome [92], Alagille syndrome [93,97,99], Turner syndrome [94], Behcet’s disease [95], Sneddon’s syndrome [96], Smith-Magenis syndrome [98], FIRES [101], PHACE syndrome [102], VACTERL association [103], Morning Glory syndrome [104], May-Hegglin anomaly [105], and mesial temporal sclerosis syndrome [106,107].

Specifically, the association between Down syndrome and moyamoya has been known for some time [211,212], with as many as 80 distinct cases, excluding large population analyses, being reported in the literature [213]. Although the pathophysiology of this association is not well understood, it might be related to developmental vascular anomalies [212], especially regarding proteins involved in vascular physiology that are encoded in chromosome 21, such as cystathionine B-synthetase, interferon-gamma receptor, superoxide dismutase, and chains of collagen type VI. Antiphospholipid antibodies have also been found in both moyamoya and Down syndrome and may reflect a possible link between these two pathologic entities [90]. Moreover, patients with Down syndrome have a higher prevalence of autoimmune disorders and autoantibodies, similar to moyamoya, which has been noted for its association with autoimmune disorders, and prevalence of antiphospholipid antibodies [16].

However, less is known about the particular Western expression and prevalence of moyamoya in association with Down syndrome [90,213,214]. In probably the first large analysis in Western cohorts, Kainth et al. calculated the prevalence of 3,760 Down syndrome diagnoses in every 100,000 moyamoya cases (approximately 3.8%) using the NIS dataset of admissions between 2002 and 2009 [16]. Given that the estimated prevalence of Down syndrome among live births from 2004 to 2006 previously calculated by Parker et al. was 14.47 per 100,000 [215], moyamoya patients are estimated to have a 26-fold increased prevalence of Down syndrome relative to the general population. Moreover, the prevalence of Down syndrome among patients admitted with moyamoya who were <15 years of age was found to be 9.5% or 9,540 per 100,000 live births. Finally, the incidence of moyamoya among Down syndrome patients was approximately three times higher than the general population [214].

There appears to be a nuanced difference between the demographic presentation of moyamoya in Down syndrome and the typical presentation. First, the percentage prevalence of Down syndrome among moyamoya patients appears to be the highest in white (65.4% vs. 47.4%) and Hispanic patients (14.6% vs. 10.4%) and especially low among blacks (10.7% vs. 25.9%). The percentage prevalence among females with Down syndrome-associated moyamoya is lower than non-Down syndrome-associated moyamoya (58.4% vs. 70.5%). Moreover, patients with moyamoya who have Down syndrome are at a greater risk for ischemic symptoms and at a lesser risk for hemorrhagic strokes (p < 0.05). On average, they experience a hospital admission at a younger age (16.2 vs. 33) [16], despite the fact that their moyamoya initially presents at an older age (8.4 vs. 6.5) [213]. These patients more often achieve better neurologic outcomes after surgical revascularization than non-Down syndrome-associated moyamoya (as reflected in improved modified Rankin scale scores in 97% of cases and lack of post-discharge strokes), despite more often being symptomatic (100% vs. 75%) and experiencing surgical complications (perioperative stroke rate per hemisphere 5.9% vs. 4%, and perioperative seizure high at 16%). Down syndrome-associated moyamoya patients are reported to have strokes more often than non-Down syndrome moyamoya patients (87% vs 68%) and more often experience seizures before diagnosis (26% vs. 6%) [90].

Demographics

The results of three studies using the NIS, the largest publicly available, all-payer inpatient database in the United States that represents 20% of all annual US hospital admissions, are presented in Table 5.

**Table 6:** Demographic Data (as %) of US Moyamoya Patients from Three NIS Studies.

Race/Admission Years	1988-2004 [17]	2002-2008 [12]	2005-2008 [14]
White	35.4	49	48.7
African American	19.7	24	24.72
Asian/Pacific Islanders	8.3	11	N/A
Hispanic	5.6	11	10.5
Native American	1.4	N/A	N/A
Other	N/A	5	15.9

Both Japanese and US literature have reported bimodal age distribution of moyamoya prevalence, with peaks in the first and third-to-fourth decades [202]. Additionally, from 1988 to 2004, African Americans accounted for the majority of patients under 19 years of age, which might be an artifact of sickle cell disease, which has a known association with moyamoya. Finally, in a study of moyamoya patients from 1987 to 1998, racial and ethnic disparities between hemispheres appeared to have been maintained in immigrant populations in the United States [186], supporting a genetic pathophysiologic etiology as the classic cause of moyamoya.

In a more recent NIS study using admission dates from 2008 to 2015, incidence of moyamoya in the US population was shown to be increasing and greatest for low-income, urban-living, female (estimated difference of 0.237, p < 0.05), and 18-44-year-old patients. Incidence rates for ethnicities were 0.509 for Asian/Pacific Islanders, 0.292 for blacks, 0.148 for whites, and 0.121 for Hispanics.

Estimates for relative rates of moyamoya between biological sexes range from 61.9% to 75% among females. However, an NIS study found that moyamoya patients younger than 18 from 1988 to 2004 displayed a male predominance before 1998 and that the rates of moyamoya among females significantly increased after 1994 for ages 36-55. Interestingly, the study also found that among African Americans, there was no significant female predominance [17].

Discussion

This paper presents the first attempt at a scoping review of the Western presentation of MMS. Moyamoya is a unique angiographic finding, characterized by stenosis of the supraclinoid internal carotid artery, middle carotid artery, anterior carotid artery, or their branches, followed by extensive collateralization of the cerebral vasculature at the base of the skull. It has historically been considered endemic to Asian populations, especially Japananese and Korean, where most cases have been found. However, a significant minority of patients in the West have presented with moyamoya findings lacking an obvious Asian heritage. Motivations for compiling this report include: (1) prevalence rate of moyamoya has been rising in recent years [14] (although possibly due to improved diagnostics and awareness [13] rather than increased prevalence of pathophysiologic mechanisms); (2) the western phenotype has been known for some time to diverge from its Asian counterpart; and (3) moyamoya has been associated with significant risk for cerebrovascular accidents. Elucidating the disease profile can provide insights into the unique pathophysiology of moyamoya and provide the groundwork for therapeutics.

We searched PubMed for case reports on moyamoya published in the last 10 years, which resulted in 725 papers. From these, we selected 357 English-language papers that reported a moyamoya association (with moyamoya as either etiology or symptom). We then selected 153 papers by excluding those reporting on patients from Asian populations. This selection was not straightforward as patients’ heritage and location of presentation were often not available. At the risk of misidentifying a few reports and in the interest of consistency, simplicity, and efficiency, we chose to categorize the patients’ East-West localization based on the location of the lead institution from which the paper was published.

Symptoms of note were, in order of prevalence in reports, neurological, stroke, hemorrhage, headache, aneurysm, dental, and ophthalmologic. Further review of the literature revealed that neurological symptoms associated with moyamoya include laryngomalacia, hypotension encephalopathy, behavioral changes, language deficits, transient psychosis, hemiplegia and movement disorders, hearing loss, and cognitive difficulties. Vascular symptoms (excluding specific markers of moyamoya, for example, occlusion of anterior, middle cerebral, and internal carotid arteries) included vertebral artery, carotid artery, ophthalmic artery, retinal artery and vein, pulmonary artery occlusions, retinal artery tortuosity, arteriovenous fistula, and vascular smooth muscle proliferation. Ophthalmologic symptoms included acute cortical blindess, ocular ischemic syndrome, and ocular malformations.

In several large analyses of multi-year NIS data, ischemic stroke was the most common symptom occurring in association with moyamoya, followed by hemorrhage, subarachnoid hemorrhage, TIA, anemia, and sickle cell disease.

Roughly two-thirds of western moyamoya patients were females, with numbers from NIS studies of moyamoya patients ranging from 62% to 72%, and a slightly higher ratio for moyamoya patients presenting for revascularization surgery (74%). The female-to-male ratio appears to be rising, with 61.9% female cases reported during 1988-2004 and 70.5% during 2002-2009.

Common disease profiles of moyamoya patients include, in order of prevalence in reports, syndromes, vascular, congenital, hematologic, neurocutaneous, autoimmune, radiation therapy, metabolic, physical trauma, drugs, and post-infectious pathologies. Vascular comorbidities were especially common, with one 2018 study of revascularization moyamoya patients reporting that one of four presented with diabetes, half with hypertension, and 40% for both hypercholesterolemia and smoking.

Of note, Down syndrome, a common condition associated with moyamoya, is more prevalent in Caucasian and Hispanic populations, and less common in women relative to the non-Down syndrome moyamoya population. Down syndrome patients with moyamoya are diagnosed with moyamoya at an older age but present at a hospital at a younger age and are more symptomatic (especially strokes and seizures), although they have better neurologic outcomes after revascularization surgery than non-Down syndrome moyamoya patients. Additionally, tumor-associated moyamoa appears to be a rare occurrence.

Autoimmune conditions commonly occur in association with moyamoya, and we found nine reports in our literature serac (10%). This is not surprising given that moyamoya has long been thought to have a pathophysiologic relationship with autoimmunity. This has been supported by half a century of research, although more recent findings have called this paradigm into question [184].

Limitations to our review include the inconsistency of reports included in our analysis, such that some reports may have been inappropriately excluded and that those included might not be representative of the overall prevalence of Western patients. Additionally, using the location of the first author as a surrogate for the patient’s heritage has obvious limitations as an immigrant or descendent of immigrants would not freely share ethnicity. Moreover, generalized disease and symptom profiles serve as only a guide for future research but cannot provide specific extrapolations for individual presentations.

## Conclusions

Our scoping review of the relevant Western literature on moyamoya has revealed an interesting panoply of demographic data, symptomatology, and associated diseases. Moyamoya is part of a complex web of interconnected disease processes that culminate in a unique radiographic finding. Moreover, while moyamoya is often thought of as native to East Asia, it presents often enough in the West that it can be understood as a unique moyamoya phenotype. Here, we aimed to collate literature reports on moyamoya presentation in the West to better appreciate and understand the unique Western moyamoya phenotype. We hope that this information guides treatment planning as moyamoya findings can herald the advent or concurrence of potentially fatal disease processes. Additionally, highlighting the unique characteristics of the Western moyamoya presentation can help further guide researchers to important discoveries in moyamoya pathophysiologic pathways and sequelae. This could, in turn, translate into discoveries of more effective treatment targets and therapeutic developments. Further research is warranted in the form of rigorously conducted systematic reviews to better contrast the Eastern and Western moyamoya phenotypes to stratify treatment planning with a greater degree of granularity.

## References

[1] Suzuki J, Takaku A (1969). Cerebrovascular "moyamoya" disease. Disease showing abnormal net-like vessels in base of brain. Arch Neurol.

[2] Kim JM, Lee SH, Roh JK (2009). Changing ischaemic lesion patterns in adult moyamoya disease. J Neurol Neurosurg Psychiatry.

[3] Suzuki J, Kodama N (1971). Cerebrovascular "moyamoya" disease: second report: collateral routes to forebrain via ethmoid sinus and superior nasal meatus. Angiology.

[4] Kamada F, Aoki Y, Narisawa A (2011). A genome-wide association study identifies RNF213 as the first moyamoya disease gene. J Hum Genet.

[5] Ito A, Fujimura M, Niizuma K (2015). Enhanced post-ischemic angiogenesis in mice lacking RNF213; a susceptibility gene for moyamoya disease. Brain Res.

[6] Ohkubo K, Sakai Y, Inoue H (2015). Moyamoya disease susceptibility gene RNF213 links inflammatory and angiogenic signals in endothelial cells. Sci Rep.

[7] Liu W, Morito D, Takashima S (2011). Identification of RNF213 as a susceptibility gene for moyamoya disease and its possible role in vascular development. PLoS One.

[8] Zhang Q, Liu Y, Yu L (2017). The association of the RNF213 p.R4810K polymorphism with quasi-moyamoya disease and a review of the pertinent literature. World Neurosurg.

[9] Milewicz DM, Østergaard JR, Ala-Kokko LM (2010). De novo ACTA2 mutation causes a novel syndrome of multisystemic smooth muscle dysfunction. Am J Med Genet A.

[10] Scott RM, Smith ER (2009). Moyamoya disease and moyamoya syndrome. N Engl J Med.

[11] Chen JB, Liu Y, Zhou LX, Sun H, He M, You C (2015). Prevalence of autoimmune disease in moyamoya disease patients in Western Chinese population. J Neurol Sci.

[12] Starke RM, Crowley RW, Maltenfort M (2012). Moyamoya disorder in the United States. Neurosurgery.

[13] Kahn A, Kaur G, Stein L, Tuhrim S, Dhamoon MS (2018). Treatment course and outcomes after revascularization surgery for moyamoya disease in adults. J Neurol.

[14] Kainth D, Chaudhry SA, Kainth H, Suri FK, Qureshi AI (2013). Epidemiological and clinical features of moyamoya disease in the USA. Neuroepidemiology.

[15] Mejia-Munne JC, Ellis JA, Feldstein NA, Meyers PM, Connolly ES (2017). Moyamoya and inflammation. World Neurosurg.

[16] Kainth DS, Chaudhry SA, Kainth HS, Suri FK, Qureshi AI (2013). Prevalence and characteristics of concurrent Down syndrome in patients with moyamoya disease. Neurosurgery.

[17] Lee DJ, Liebeskind DS (2011). Characterization of inpatient moyamoya in the United States: 1988-2004. Front Neurol.

[18] Ghaffari-Rafi A, Ghaffari-Rafi S, Leon-Rojas J (2020). Socioeconomic and demographic disparities of moyamoya disease in the United States. Clin Neurol Neurosurg.

[19] Hong JC, Ramos E, Copeland CC, Ziv K (2016). Transient intraoperative central diabetes insipidus in moyamoya patients undergoing revascularization surgery: a mere coincidence?. A A Case Rep.

[20] Romão EA, Lourenço CM, Júnior WM (2013). What lies beneath: Fabry nephropathy in a female patient with severe cerebrovascular disease. Clin Nephrol.

[21] Unger MD, Georges J, Shaikh HA, Kavi T (2018). Moyamoya tipping point: fatal bilateral MCA territory infarction following cocaine abuse. BMJ Case Rep.

[22] Ullrich NJ, Zimmerman M, Smith E, Irons M, Marcus K, Kieran MW (2011). Association of rapidly progressive moyamoya syndrome with bevacizumab treatment for glioblastoma in a child with neurofibromatosis type 1. J Child Neurol.

[23] Zhuo DX, Ragosta M III, Patterson B (2019). Tyrosine kinase inhibitor toxicity manifesting as comorbid moyamoya syndrome and obstructive coronary artery disease: a case report and review of the literature. Catheter Cardiovasc Interv.

[24] Budişteanu M, Burloiu CM, Papuc SM, Focşa IO, Riga D, Riga S, Arghir A (2019). Neurofibromatosis type 1 associated with moyamoya syndrome. Case report and review of the literature. Rom J Morphol Embryol.

[25] Delvoye F, Hervé D, Chabriat H, Mawet J (2013). Moyamoya syndrome related to neurofibromatosis of type 1: a case report. Acta Neurol Belg.

[26] Novara S, Singh S, Rashid S (2017). "Ivy sign" and moyamoya disease in a child with neurofibromatosis type 1. Pediatr Neurol.

[27] Vargiami E, Sapountzi E, Samakovitis D, Batzios S, Kyriazi M, Anastasiou A, Zafeiriou DI (2014). Moyamoya syndrome and neurofibromatosis type 1. Ital J Pediatr.

[28] Mayl J, Patel H, Chandra T (2017). Moyamoya syndrome in a child with neurofibromatosis type 1: magnetic resonance imaging as a tool for clinical decision making. Cureus.

[29] Serafini NB, Serafini CB, Vinhas AS, Godinho MB (2017). Moyamoya syndrome associated with neurofibromatosis type 1 in a pediatric patient. An Bras Dermatol.

[30] Jiménez Caballero PE (2016). [Adult-onset moyamoya disease in a patient with neurofibromatosis type 1]. Neurologia.

[31] Gold JJ, Dory CE, Levy ML, Crawford JR (2013). Simultaneous moyamoya disease and cervical spinal cord low-grade astrocytoma in a child with neurofibromatosis type 1. BMJ Case Rep.

[32] Darrigo Júnior LG, Valera ET, Machado Ade A, Santos AC, Scrideli CA, Tone LG (2011). Moyamoya syndrome associated with neurofibromatosis type I in a pediatric patient. Sao Paulo Med J.

[33] Smith M, Heran MK, Connolly MB (2011). Cerebrovasculopathy in NF1 associated with ocular and scalp defects. Am J Med Genet A.

[34] Golomb MR, Smith JL (2014). Poor wound healing after pial synangiosis in 2 children with moyamoya vasculopathy associated with neurofibromatosis type 1. J Child Neurol.

[35] Lai J, Modi L, Ramai D, Tortora M (2017). Tuberous sclerosis complex and polycystic kidney disease contiguous gene syndrome with moyamoya disease. Pathol Res Pract.

[36] de Guzman P, Hamilton M, Khan A, Eesa M, Kirton A (2014). Moyamoya syndrome associated with mucolipidosis-II. Can J Neurol Sci.

[37] Çevik B, Acu B, Aksoy D, Kurt S (2014). Protein S deficiency and an adult case with moyamoya syndrome that presented with primary intraventricular haemorrhage. Balkan Med J.

[38] Klemann C, Kirschner J, Ammann S (2018). CD59 deficiency presenting as polyneuropathy and moyamoya syndrome with endothelial abnormalities of small brain vessels. Eur J Paediatr Neurol.

[39] Cho SM, Di Lorenzo R, Myles JL, Uchino K (2018). Severe hyperhomocysteinemia manifesting as moyamoya vasculopathy and Henoch-Schonlein purpura. Neurology.

[40] Ghilain V, Wiame E, Fomekong E, Vincent MF, Dumitriu D, Nassogne MC (2016). Unusual association between lysinuric protein intolerance and moyamoya vasculopathy. Eur J Paediatr Neurol.

[41] Zaletel M, Surlan-Popović K, Pretnar-Oblak J, Zvan B (2011). Moyamoya syndrome with arteriovenous dural fistula after head trauma. Acta Clin Croat.

[42] Dezmalj-Grbelja L, Bosnjak J, Lovrencić-Huzjan A, Ivica M, Demarin V (2010). Moyamoya disease in a patient with brain tumor: case report. Acta Clin Croat.

[43] Lang M, Moore NZ, Witek AM, Kshettry VR, Bain MD (2017). Microsurgical repair of ruptured aneurysms associated with moyamoya-pattern collateral vessels of the middle cerebral artery: a report of two cases. World Neurosurg.

[44] Choi J, Suthakar P, Farmand F (2016). Fatal outcome in a Hispanic woman with moyamoya syndrome and Graves’ disease. Endocrinol Diabetes Metab Case Rep.

[45] Sakalli H, Baskin E, Alehan F, Agıldere M, Akova YA, Caner H (2012). Dual anca positivity in a child with moyamoya-like cerebral vascular changes: an unusual presentation with sudden homonymous hemianopsia. Rheumatol Int.

[46] Ahmad AS, Tahir RA, Mitsias PD (2018). Moyamoya vasculopathy with anti-SCL-70 antibodies: a case report and review of the literature. J Clin Neurosci.

[47] Wegner F, Müller-Ladner U, Meier FM (2019). Reply to 'Moyamoya vasculopathy with anti-SCL-70 antibodies: A case report and review of the literature'. J Clin Neurosci.

[48] Troedson C, Wong M, Dalby-Payne J (2013). Systemic lupus erythematosus due to C1q deficiency with progressive encephalopathy, intracranial calcification and acquired moyamoya cerebral vasculopathy. Lupus.

[49] Valluri M, Akhondi H, Hyndman M (2018). Moyamoya complicated by thrombotic cerebrovascular accident in a Caucasian woman with collagenous colitis. Neurol Sci.

[50] Wegner F, Mueller-Ladner U, Meier FM (2016). Moyamoya disease and systemic sclerosis (MoSys syndrome): a combination of two rare entities: comment to the authors. Clin Exp Rheumatol.

[51] Skeik N, Rumery KK, Udayakumar PD, Crandall BM, Warrington KJ, Sullivan TM (2013). Concurrent Takayasu arteritis with common variable immunodeficiency and moyamoya disease. Ann Vasc Surg.

[52] Hughes JW, Wyckoff JA, Hollander AS, Derdeyn CP, McGill JB (2016). Moyamoya syndrome causing stroke in young women with type 1 diabetes. J Diabetes Complicat.

[53] Malik S, Russman AN, Katramados AM, Silver B, Mitsias PD (2011). Moyamoya syndrome associated with Graves' disease: a case report and review of the literature. J Stroke Cerebrovasc Dis.

[54] Brandicourt P, Bonnet L, Béjot Y, Drouet C, Moulin T, Thines L (2018). Moya-Moya syndrome after cranial radiation for optic glioma with NF1. Case report and literature review of syndromic cases. Neurochirurgie.

[55] Reynolds MR, Haydon DH, Caird J, Leonard JR (2016). Radiation-induced moyamoya syndrome after proton beam therapy in the pediatric patient: a case series. Pediatr Neurosurg.

[56] Manion B, Sung WS (2011). Radiation-induced moyamoya disease after childhood astrocytoma. J Clin Neurosci.

[57] Zwagerman NT, Foster K, Jakacki R, Khan FH, Yock TI, Greene S (2014). The development of moyamoya syndrome after proton beam therapy. Pediatr Blood Cancer.

[58] Scala M, Vennarini S, Garrè ML (2019). Radiation-induced moyamoya syndrome after proton therapy in child with clival chordoma: natural history and surgical treatment. World Neurosurg.

[59] Almeida P, Rocha AL, Alves G (2019). Moyamoya syndrome after radiation therapy: a clinical report. Eur J Case Rep Intern Med.

[60] Guey S, Grangeon L, Brunelle F (2017). De novo mutations in CBL causing early-onset paediatric moyamoya angiopathy. J Med Genet.

[61] Bayram AK, Yilmaz E, Per H (2016). Familial moyamoya disease in two Turkish siblings with same polymorphism in RNF213 gene but different clinical features. Childs Nerv Syst.

[62] Luisa SF, Rizzo A, Bedini G (2018). Microduplication of 15q13.3 and microdeletion of 18q21.32 in a patient with moyamoya syndrome. Int J Mol Sci.

[63] Keylock A, Hong Y, Saunders D (2018). Moyamoya-like cerebrovascular disease in a child with a novel mutation in myosin heavy chain 11. Neurology.

[64] Toldo I, Po C, Morao V (2016). Moyamoya syndrome and 6p chromosome rearrangements: expanding evidences of a new association. Eur J Paediatr Neurol.

[65] Melis M, Cau M, Corraine S, Secci S, Addis M, Melis M (2014). Cerebral cavernous malformations and unilateral moyamoya in a patient with a new mutation in the KRIT-1/CCM1 gene. Cerebrovasc Dis.

[66] Wallace S, Guo DC, Regalado E (2016). Disrupted nitric oxide signaling due to GUCY1A3 mutations increases risk for moyamoya disease, achalasia and hypertension. Clin Genet.

[67] Schilter KF, Steiner JE, Demos W (2017). RNF213 variants in a child with PHACE syndrome and moyamoya vasculopathy. Am J Med Genet A.

[68] Harel T, Posey JE, Graham BH, Walkiewicz M, Yang Y, Lalani SR, Belmont JW (2015). Atypical presentation of moyamoya disease in an infant with a de novo RNF213 variant. Am J Med Genet A.

[69] Teo M, Johnson JN, Bell-Stephens TE (2016). Surgical outcomes of Majewski osteodysplastic primordial dwarfism type II with intracranial vascular anomalies. J Neurosurg Pediatr.

[70] Kılıç E, Utine E, Unal S (2012). Medical management of moyamoya disease and recurrent stroke in an infant with Majewski osteodysplastic primordial dwarfism type II (MOPD II). Eur J Pediatr.

[71] Bang GM, Kirmani S, Patton A, Pulido JS, Brodsky MC (2013). “Ocular moyamoya” syndrome in a patient with features of microcephalic osteodysplastic primordial dwarfism type II. J AAPOS.

[72] Prontera P, Rogaia D, Mencarelli A (2017). Juvenile moyamoya and craniosynostosis in a child with deletion 1p32p31: expanding the clinical spectrum of 1p32p31 deletion syndrome and a review of the literature. Int J Mol Sci.

[73] Vo Van P, Sabouraud P, Mac G, Abely M, Bednarek N (2011). Moyamoya disease associated with hereditary spherocytosis. Pediatr Neurol.

[74] Yadegari S, Aminian A (2017). Management in rare association of moyamoya syndrome and hereditary spherocytosis. Minerva Pediatr.

[75] Gait-Carr E, Connolly DJ, King D (2017). Moyamoya syndrome associated with hereditary spherocytosis: an emerging clinical entity. J Pediatr Hematol Oncol.

[76] Karvandian K, Khan ZH, Zebardast J, Miri SR (2011). Laparoscopic splenectomy in a child with moyamoya syndrome, hereditary spherocytosis, and interstitial lung disease: a mere coincidence or partnership based on genetic similarities. Case Rep Anesthesiol.

[77] Ray A, Rodriguez N (2011). Cerebral infarction in the setting of moyamoya in a pediatric patient with sickle β+-thalassemia. Pediatr Hematol Oncol.

[78] Al-Hawsawi ZM, Al-Zaid MA, Barnawi AI, Yassine SM (2015). Fanconi anemia associated with moyamoya disease in Saudi Arabia. Saudi Med J.

[79] Soares D, Bullock R, Ali S (2014). Moyamoya syndrome in sickle cell anaemia: a cause of recurrent stroke. BMJ Case Rep.

[80] Janczar S, Fogtman A, Koblowska M (2014). Novel severe hemophilia A and moyamoya (SHAM) syndrome caused by Xq28 deletions encompassing F8 and BRCC3 genes. Blood.

[81] Delavari N, Strahle J, Maher CO (2013). Moyamoya syndrome associated with hemoglobin Southampton (Casper). Pediatr Neurosurg.

[82] Inati A, Tourjuman O, Bizri D (2013). A rare case of Moyamoya syndrome in a β-thalassemia major patient. Blood Cells Mol Dis.

[83] Ebert JJ, Sisk RA (2019). CRAO in moyamoya syndrome associated with Southampton hemoglobinopathy. Ophthalmic Surg Lasers Imaging Retina.

[84] Lavin M, Jenkins PV, Keenan C, White B, Betts DR, O'Donnell JS, O'Connell NM (2016). X-linked moyamoya syndrome associated with severe haemophilia A. Haemophilia.

[85] Komur M, Unal S, Okuyaz C, Ozgur A (2014). Moyamoya syndrome associated with sickle cell trait in a child. Brain Dev.

[86] Agrawal R, Berube C, Steinberg G, George TI (2013). Moyamoya syndrome with sickle cell trait. Int J Lab Hematol.

[87] El Beltagi AH, El-Sheikh A, El-Saif R, Norbash A (2014). Ivy sign in mildly symptomatic β-thalassemia intermedia, with development of moyamoya disease. Neuroradiol J.

[88] Shiari R, Tabatabaei Nodushan SM, Mohebbi MM, Karimzadeh P, Javadzadeh M (2016). Moyamoya syndrome associated with Henoch-Schönlein purpura. Iran J Child Neurol.

[89] Horev L, Lees MM, Anteby I, Gomori JM, Gunny R, Ben-Neriah Z (2011). Oculoectodermal syndrome with coarctation of the aorta and moyamoya disease: expanding the phenotype to include vascular anomalies. Am J Med Genet A.

[90] Tavares Bello C, Barreiros C, Gil I, Vasconcelos C (2017). Down syndrome and moyamoya disease: unusual cause of stroke. BMJ Case Rep.

[91] Gupta M, Choudhri OA, Feroze AH, Do HM, Grant GA, Steinberg GK (2016). Management of moyamoya syndrome in patients with Noonan syndrome. J Clin Neurosci.

[92] Cullu N, Karakas E, Karakas O, Deveer M, Calik M, Boyaci FN (2013). Childhood moyamoya disease accompanying Leigh syndrome. J Pak Med Assoc.

[93] Rocha R, Soro I, Leitão A, Silva ML, Leão M (2012). Moyamoya vascular pattern in Alagille syndrome. Pediatr Neurol.

[94] Manjila S, Miller BR, Rao-Frisch A, Otvos B, Mitchell A, Bambakidis NC, De Georgia MA (2014). Moyamoya disease associated with asymptomatic mosaic Turner syndrome: a rare cause of hemorrhagic stroke. J Stroke Cerebrovasc Dis.

[95] Degirmenci E, Bir LS, Yagcı B, Nazliel B, Siva A (2014). Moyamoya syndrome or Behçet's disease?. Int J Rheum Dis.

[96] Fierini F, Barilaro A, Giambene B, Carlucci G, Grandi V, Maio V, Pantoni L (2015). Moyamoya in a patient with Sneddon's syndrome. Clin Neurol Neurosurg.

[97] Delaney S, O'Connor G, Reardon W (2018). Extracranial and intracranial vasculopathy with “moyamoya phenomenon” in association with Alagille syndrome. Front Neurol.

[98] Freeman J, Deleyiannis F, Bernard TJ, Fenton LZ, Somme S, Wilkinson CC (2017). Moyamoya in a patient with Smith-Magenis syndrome. Pediatr Neurosurg.

[99] Pavanello M, Severino M, D'Antiga L, Castellan L, Calvi A, Colledan M, Gandolfo C (2015). Pretransplant management of basilar artery aneurysm and moyamoya disease in a child with Alagille syndrome. Liver Transpl.

[100] Vimalesvaran S, Nachiappan N, Sithamparanathan Y (2013). Moyamoya syndrome in a Malaysian child with Down syndrome. J Paediatr Child Health.

[101] Kaufman T, White A (2017). Moyamoya in a patient with FIRES: a first case report. Neurodiagn J.

[102] Martel C, Robertson R, Williams FB, Moore RC, Clark A (2015). Anesthetic management of a parturient with PHACE syndrome for cesarean delivery. A A Case Rep.

[103] Hong CS, Wang AC, Bonow RH, Abecassis IJ, Amlie-Lefond C, Ellenbogen RG (2016). Moyamoya disease in a patient with VACTERL association. World Neurosurg.

[104] Nezzar H, Mbekeani JN, Dalens H (2015). Morning glory syndrome with carotid and middle cerebral artery casculopathy. Optom Vis Sci.

[105] Béjot Y, Barnay JL, Chavent A (2017). Subarachnoid hemorrhage revealing moyamoya syndrome in a patient with May-Hegglin anomaly. Neurologist.

[106] Gross BA, Ropper AE, Du R (2013). Association of mesial temporal sclerosis and moyamoya syndrome. Clin Neurol Neurosurg.

[107] Lohani S, Madsen JR, Bergin AM, Smith ER (2013). Moyamoya disease with mesial temporal sclerosis. J Neurosurg Pediatr.

[108] Diaz UJ, Cabán-Martinez AJ, Halder GE (2014). Presentation with recurrent intractable headache: a patient with moyamoya syndrome: case report. Neurol Med Chir (Tokyo).

[109] Azzam DB, Sharma AN, Tiourin E, Chan AY (2019). A case report of moyamoya disease presenting as headache in a 35-year-old Hispanic man. Cureus.

[110] Elia M (2018). Bilateral visual field loss in an adolescent girl with migraine headaches. JAMA Ophthalmol.

[111] Verdure P, Le Moigne O, Massardier EG, Vanhulle C, Tollard E, Maltête D (2012). [Migraine-like headache and ocular malformations may herald moyamoya syndrome]. Rev Neurol (Paris).

[112] Tozzi E, Antenucci A, Di Loreto S, Maresca M, Farello G, Massimi L (2015). Moyamoya disease and headache: case report. J Headache Pain.

[113] Pinardi F, Stracciari A, Spinardi L, Guarino M (2013). Postpneumococcal moyamoya syndrome case report and review of the postinfective cases. BMJ Case Rep.

[114] Trombatore P, Lozupone E, Gaudino S (2020). A rare case of postinfectious moyamoya syndrome: case report and review of the literature. World Neurosurg.

[115] Sajja A, Tsering D, Mooser AC, DeFreitas TA, Carpenter J, Magge SN (2017). Patient with severe moyamoya disease who presents with acute cortical blindness. Stroke.

[116] Papavasileiou E, Sobrin L, Papaliodis GN (2015). Ocular ischemic syndrome presenting as retinal vasculitis in a patient with moyamoya syndrome. Retin Cases Brief Rep.

[117] Santos AF, Pinho J, Ferreira C, Pereira C, Ribeiro M, Rocha J (2014). "The fainted man:" hypoperfusion encephalopathy in a patient with moyamoya. J Neuropsychiatry Clin Neurosci.

[118] Born H, Wineland A, Rutter MJ (2019). Neurologically acquired laryngomalacia in a pediatric patient with moyamoya: a case report and literature review. Int J Pediatr Otorhinolaryngol.

[119] Lai J, Patel A, Dandurand C, Gooderham P, Lu S (2018). Depression and catatonia: a case of neuropsychiatric complications of moyamoya disease. Cureus.

[120] Indorewalla KK, McArdle M, Tomlinson E, Piryatinsky I (2020). Neuropsychological profile associated with moyamoya disease: a case report. NeuroRehabilitation.

[121] Richards M, Grzenda A, Nelson E, Gitlin M (2019). Psychiatric comorbidity in moyamoya disease and preliminary guidelines for treatment. Am J Psychiatry.

[122] Hamamoto Filho PT, Lira CCS, Zanini MA (2018). Moyamoya syndrome manifesting with choreiform movements. Neuropediatrics.

[123] Platzen J, Berlit P, Kraemer M (2017). Chorea gravidarum associated with moyamoya angiopathy treated with alpha-methyldopa. Clin Neurol Neurosurg.

[124] Laatar F, Kacem I, Nasri A, Ben Djebara M, Gargouri A, Gouider R (2017). Generalized dystonia: unusual mode of revelation of moyamoya disease. Tunis Med.

[125] Greene S, Bansal L, Coffman KA, Nardone R, Zuccoli G (2016). Pial synangiosis ameliorates movement disorders in the absence of prior stroke in moyamoya disease. J Child Neurol.

[126] Tam HH, Amiras D, Patel M, Win Z (2013). An unusual cause of hemiplegia in a 28-year-old woman. Br J Hosp Med (Lond).

[127] Pandey P, Bell-Stephens T, Steinberg GK (2010). Patients with moyamoya disease presenting with movement disorder. J Neurosurg Pediatr.

[128] Lamônica DA, Ribeiro CD, Ferraz PM, Tabaquim ML (2016). [Moyamoya disease: impact on the performance of oral and written language]. Codas.

[129] Zalonis I, Christidi F, Kararizou E, Triantafyllou NI, Spengos K, Vassilopoulos D (2010). Cognitive deficits presenting as psychiatric symptoms in a patient with moyamoya disease. Psychol Rep.

[130] Wu TY, Smith A, Kilfoyle D (2013). Hemifacial spasm leading to diagnosis of moyamoya disease. N Z Med J.

[131] Bidaki R, Zarepur E (2017). Intermittent hemiplegia in a boy with primary moyamoya disease: a case report from Iran. Iran J Child Neurol.

[132] Gül F, Berçin S, Müderris T, Yalçıner G, Ünal Ö, Kırış M (2016). Unilateral sudden hearing loss: a rare symptom of moyamoya disease. Kulak Burun Bogaz Ihtis Derg.

[133] Schranz D, Kerst G, Menges T (2015). Transcatheter creation of a reverse Potts shunt in a patient with severe pulmonary arterial hypertension associated with moyamoya syndrome. Eurointervention.

[134] Kızılkaya MH, Uysal F, Gürbüz E, Taşkapılıoğlu M, Bostan ÖM (2018). Atypical presentation of moyamoya disease with pulmonary hypertension: a case report. Anatol J Cardiol.

[135] Migliore F, Iorember FM, Gedalia A (2018). 6-year-old with severe hypertension. Clin Pediatr (Phila).

[136] Witmer MT, Levy R, Yohay K, Kiss S (2013). Ophthalmic artery ischemic syndrome associated with neurofibromatosis and moyamoya syndrome. JAMA Ophthalmol.

[137] Savio K, Mittino D, Terazzi E, Divenuto I, Fossaceca R, Carriero A, Monaco F (2010). Moyamoya disease and arteriovenous fistula of the epiaortic vessels. Neurol Sci.

[138] Karsten MB, Oliveira C, Segal AZ, Scott RM (2020). Central retinal artery occlusion occurring 30 years after successful revascularization surgery for moyamoya disease: case report. Acta Neurochir (Wien).

[139] Katsman D, Klufas MA, Sarraf D, Sadda S (2016). Retinal arterial tortuosity in moyamoya disease. JAMA Ophthalmol.

[140] Güçlü H, Gurlu VP, Ozal SA, Esgin H (2016). A moyamoya patient with bilateral consecutive branch retinal vein occlusion. Neuroophthalmology.

[141] Jiang T, Perry A, Dacey RG, Jr. Jr., Zipfel GJ, Derdeyn CP (2013). Intracranial atherosclerotic disease associated with moyamoya collateral formation: histopathological findings. J Neurosurg.

[142] Toscano M, Puledda F, Viganò A, Vicenzini E, Guidetti G, Lenzi GL, Di Piero V (2014). Hemodynamic features of non-aneurysmal subarachnoid hemorrhage in a case of familial moyamoya disease: a transcranial Doppler ultrasound study. Eur Neurol.

[143] Beyaz P, Khan N, Baltsavias G (2018). Multiple anomalies in the origin and course of vertebral arteries and aberrant right subclavian artery in a child with moyamoya syndrome. BMJ Case Rep.

[144] Lutz T, Mönnings P, Ayzenberg I, Lukas C (2018). Twig-like middle cerebral artery: a seldom vessel anomaly of important relevance. Clin Neuroradiol.

[145] Reid AJ, Bhattacharjee MB, Regalado ES, Milewicz AL, El-Hakam LM, Dauser RC, Milewicz DM (2010). Diffuse and uncontrolled vascular smooth muscle cell proliferation in rapidly progressing pediatric moyamoya disease. J Neurosurg Pediatr.

[146] Garoon R, Carvounis PE (2015). Central retinal vein occlusion with bilateral stenosis of the internal carotid arteries. Lancet.

[147] Moceri P, Laïk J, Bouvaist H, Fraisse A, Ferrari E (2016). Peripheral pulmonary artery stenoses in the setting of moyamoya. Eur Heart J Cardiovasc Imaging.

[148] Rajanala AP, Le HT, Gill MK (2020). Central retinal artery occlusion as initial presentation of moyamoya disease in a middle-aged woman. Am J Ophthalmol Case Rep.

[149] Koduri S, Wilkinson DA, Griauzde JM, Gemmete JJ, Maher CO (2019). Development of bilateral dural arteriovenous fistulae following pial synangiosis for moyamoya syndrome: case report. J Neurosurg Pediatr.

[150] Livesay J, Johnson J (2019). ST-elevation myocardial infarction (STEMI) in a patient with moyamoya disease. Case Rep Cardiol.

[151] Kraemer M, Diehl RR, Diesner F, Berlit P, Khan N (2012). Differential diagnosis between cerebral ischemia, focal seizures and limb shaking TIAs in moyamoya disease. Br J Neurosurg.

[152] Ozer M, Merchant K, Manning Z, Goksu SY, Juneja K, Fennell VS (2020). Postpartum-onset moyamoya disease: a rare cause of stroke in unexpected. Case Rep Neurol Med.

[153] Chaughtai S, Chaughtai Z, Haider MS (2019). Subacute stroke in a young female: a case of moyamoya syndrome initially anchoring with anxiety. Case Rep Med.

[154] Ilyayeva E, Nada K, Farahi Far R, Albright K, Gujral MK, Gold M (2018). Bilateral cerebrovascular stroke as an initial presenting symptom of moyamoya disease. Case Rep Crit Care.

[155] Mohammadi O, Krieger D, Butt I, Danckers M (2019). A case of delayed diagnosis of moyamoya disease after recurrent strokes. Cureus.

[156] Omer S, Zbyszynska R, Kirthivasan R (2018). Peek through the smoke: a report of moyamoya disease in a 32-year-old female patient presenting with ischaemic stroke. BMJ Case Rep.

[157] Casserly CS, Salmon A, Ramsay DA, Pelz DM, Lownie SP, Strong MJ (2012). Multiple cerebral infarcts in patient with moyamoya disease. Can J Neurol Sci.

[158] Djedovic G, Verstappen R, Matiasek J, Engelhardt TO, Pierer G, Rieger UM (2014). Occult moyamoya disease causing fulminant infarction after septorhinoplasty. Case Rep Plast Surg Hand Surg.

[159] Bechan RS, van Rooij WJ (2014). Endovascular treatment of a ruptured flow aneurysm of the heubner artery as part of a moyamoya collateral network in a young patient with an occluded middle cerebral artery. Interv Neuroradiol.

[160] Heijmen L, van Dijk EJ, Goraj B, van Laarhoven HW (2012). Moyamoya disease misdiagnosed as leptomeningeal metastases. J Clin Oncol.

[161] Mélot A, Chazot JV, Troude L, De la Rosa S, Brunel H, Roche PH (2016). [Ruptured posterior ethmoidal artery aneurysm and Moyamoya disease in an adult patient. Case report]. Neurochirurgie.

[162] Harreld JH, Zomorodi AR (2011). Embolization of an unruptured distal lenticulostriate aneurysm associated with moyamoya disease. Am J Neuroradiol.

[163] Abuoliat ZA, AlFarhan BA, Alshahrani AA, AlFarhan AA, Almuntashri MA, Alotaibi N (2017). Atypical location of intracerebral hemorrhage in moyamoya disease. Cureus.

[164] Alcalá-Cerra GA, Moscote-Salazar LR, Barrios RS, Niño-Hernández LM, Gutiérrez Paternina JJ (2011). Non-aneurysmal subarachnoid hemorrhage as presentation of moyamoya disease in an adult. Surg Neurol Int.

[165] Noureldine MH, Saikali I, Nassif A, Chahinian R, Sweid A, Kikano R, Mawad M (2020). Pediatric moyamoya presenting as a subarachnoid hemorrhage from a ruptured anterior cerebral artery aneurysm. World Neurosurg.

[166] Donohue MM, Moore A, Shibata D, Ebel-Caswell S, Becker KJ (2010). Transcranial Doppler ultrasound CO2 challenge complicated by subarachnoid hemorrhage in patient with moyamoya syndrome. Neurocrit Care.

[167] Varanasi LC, Brown J, Athayde N (2019). Postpartum seizure and subarachnoid haemorrhage secondary to moyamoya disease. Case Rep Obstet Gynecol.

[168] Plans Galván O, Manciño Contreras JM, Coy Serrano A, Campos Gómez A, Toboso Casado JM, Ricart Martí P (2019). [Intraparenchymal haemorrhage secondary to moyamoya disease in a white patient]. Neurologia (Engl Ed).

[169] Schödel P, Brawanski A, Friedrich M, Schlachetzki F, Heiss P, Schebesch KM (2013). Fatal hemorrhagic stroke in a Caucasian girl with moyamoya disease. Childs Nerv Syst.

[170] Ko BL, Unkel JH (2018). Dental management of a pediatric patient with moyamoya syndrome: a rare clinical entity. Pediatr Dent.

[171] Bo H, Avenetti D, Kratunova E (2017). Dental management considerations in a pediatric patient with moyamoya disease. J Dent Child (Chic).

[172] Kraemer M, Lee SI, Ayzenberg I (2017). Headache in Caucasian patients with moyamoya angiopathy - a systematic cohort study. Cephalalgia.

[173] Klasen H, Britton J, Newman M (1999). Moyamoya disease in a 12-year-old Caucasian boy presenting with acute transient psychosis. Eur Child Adolesc Psychiatry.

[174] Kraemer M, Trakolis L, Platzen J (2017). Movement symptoms in European moyamoya angiopathy - first systematic questionnaire study. Clin Neurol Neurosurg.

[175] Scott RM, Smith JL, Robertson RL, Madsen JR, Soriano SG, Rockoff MA (2004). Long-term outcome in children with moyamoya syndrome after cranial revascularization by pial synangiosis. J Neurosurg.

[176] Setzen G, Cacace AT, Eames F (1999). Central deafness in a young child with Moyamoya disease: paternal linkage in a Caucasian family: two case reports and a review of the literature. Int J Pediatr Otorhinolaryngol.

[177] Arias EJ, Derdeyn CP, Dacey RG, Jr. Jr., Zipfel GJ (2014). Advances and surgical considerations in the treatment of moyamoya disease. Neurosurgery.

[178] Stejskal V, Šteiner I, Hornychová H, Krůpa P, Kanta M (2020). Moyamoya disease associated with fibromuscular dysplasia of intrapulmonary bronchial arteries-a case report. Cardiovasc Pathol.

[179] Jansen JN, Donker AJ, Luth WJ, Smit LM (1990). Moyamoya disease associated with renovascular hypertension. Neuropediatrics.

[180] Kim SJ, Heo KG, Shin HY (2010). Association of thyroid autoantibodies with moyamoya-type cerebrovascular disease: a prospective study. Stroke.

[181] El Ramahi KM, Al Rayes HM (2000). Systemic lupus erythematosus associated with moyamoya syndrome. Lupus.

[182] Wang CY, Grupke SL, Roberts J, Lee J, Fraser JF (2018). Factors associated with moyamoya syndrome in a Kentucky regional population. J Stroke Cerebrovasc Dis.

[183] Hall JG, Flora C, Scott CI, Jr. Jr., Pauli RM, Tanaka KI (2004). Majewski osteodysplastic primordial dwarfism type II (MOPD II): natural history and clinical findings. Am J Med Genet A.

[184] Kronenburg A, Braun KP, van der Zwan A, Klijn CJ (2014). Recent advances in moyamoya disease: pathophysiology and treatment. Curr Neurol Neurosci Rep.

[185] Brancati F, Castori M, Mingarelli R, Dallapiccola B (2005). Majewski osteodysplastic primordial dwarfism type II (MOPD II) complicated by stroke: clinical report and review of cerebral vascular anomalies. Am J Med Genet A.

[186] Bober MB, Khan N, Kaplan J, Lewis K, Feinstein JA, Scott CI Jr., Steinberg GK (2010). Majewski osteodysplastic primordial dwarfism type II (MOPD II): expanding the vascular phenotype. Am J Med Genet A.

[187] Rahme R, Crevier L, Dubois J, Mercier C (2010). Moyamoya-like vasculopathy and Seckel syndrome: just a coincidence?. Childs Nerv Syst.

[188] Gunesli A, Andic C, Alkan O, Erol I, Suner HI (2018). Endovascular treatment of a patient with moyamoya disease and Seckel syndrome: a case report. J Pediatr Neurosci.

[189] Stowe RC, Jimenez-Gomez A, Balasa A, Clark GD (2018). Cockayne syndrome complicated by moyamoya vasculopathy and stroke. Pediatr Neurol.

[190] Coughlin DJ, Miller CA, Schuette AJ (2017). Treatment of moyamoya disease and unruptured intracranial aneurysm in floating-harbor syndrome. World Neurosurg.

[191] Sunder TR (1981). Moyamoya disease in a patient with type I glycogenosis. Arch Neurol.

[192] Baird LC, Smith ER, Ichord R (2015). Moyamoya syndrome associated with Alagille syndrome: outcome after surgical revascularization. J Pediatr.

[193] Spengos K, Kosmaidou-Aravidou Z, Tsivgoulis G, Vassilopoulou S, Grigori-Kostaraki P, Zis V (2006). Moyamoya syndrome in a Caucasian woman with Turner's syndrome. Eur J Neurol.

[194] Barrit S (2018). [An Aicardi-Goutières syndrome associated with a quasi-Moyamoya by a biallelic mutation in SAMHD1]. Rev Med Brux.

[195] Sequeira W, Naseem M, Bouffard DA (1984). An association with birth control pills. Moyamoya. IMJ Ill Med J.

[196] Peerless SJ (1997). Risk factors of moyamoya disease in Canada and the USA. Clin Neurol Neurosurg.

[197] Uchino K, Johnston SC, Becker KJ, Tirschwell DL (2005). Moyamoya disease in Washington State and California. Neurology.

[198] Merkel KH, Ginsberg PL, Parker JC, Jr. Jr., Post MJ (1978). Cerebrovascular disease in sickle cell anemia: a clinical, pathological and radiological correlation. Stroke.

[199] Newman S, Boulter JH, Malcolm JG, Pradilla I, Pradilla G (2020). Outcomes in patients with moyamoya syndrome and sickle cell disease: a systematic review. World Neurosurg.

[200] Cheng ZJ, Shen YY, Warsame IM, Dai TM, Tu JL (2018). Moyamoya syndrome caused by paroxysmal nocturnal hemoglobinuria. Chin Med J (Engl).

[201] Marden FA, Putman CM, Grant JM, Greenberg J (2008). Moyamoya disease associated with hemoglobin Fairfax and beta-thalassemia. Pediatr Neurol.

[202] Parker TM, Ward LM, Johnston DL, Ventureya E, Klaassen RJ (2009). A case of moyamoya syndrome and hemoglobin E/beta-thalassemia. Pediatr Blood Cancer.

[203] Xu F, Tang H, Xiong J, Liu X (2018). Moyamoya disease associated with tuberculum sellae meningioma and cavernous sinus hemangioma. World Neurosurg.

[204] Morgello S, Laufer H (1989). Quaternary neurosyphilis in a Haitian man with human immunodeficiency virus infection. Hum Pathol.

[205] Palacio S, Hart RG, Vollmer DG, Kagan-Hallet K (2003). Late-developing cerebral arteropathy after pyogenic meningitis. Arch Neurol.

[206] Hammond CK, Shapson-Coe A, Govender R (2016). Moyamoya syndrome in South African children with HIV-1 infection. J Child Neurol.

[207] Sharfstein SR, Ahmed S, Islam MQ, Najjar MI, Ratushny V (2007). Case of moyamoya disease in a patient with advanced acquired immunodeficiency syndrome. J Stroke Cerebrovasc Dis.

[208] Desai SS, Paulino AC, Mai WY, Teh BS (2006). Radiation-induced moyamoya syndrome. Int J Radiat Oncol Biol Phys.

[209] Servo A, Puranen M (1978). Moyamoya syndrome as a complication of radiation therapy. Case report. J Neurosurg.

[210] Kestle JR, Hoffman HJ, Mock AR (1993). Moyamoya phenomenon after radiation for optic glioma. J Neurosurg.

[211] Fukushima Y, Kondo Y, Kuroki Y, Miyake S, Iwamoto H, Sekido K, Yamaguchi K (1986). Are Down syndrome patients predisposed to moyamoya disease?. Eur J Pediatr.

[212] Fukuyama Y, Osawa M, Kanai N (1992). Moyamoya disease (syndrome) and the Down syndrome. Brain Dev.

[213] See AP, Ropper AE, Underberg DL, Robertson RL, Scott RM, Smith ER (2015). Down syndrome and moyamoya: clinical presentation and surgical management. J Neurosurg Pediatr.

[214] Rison RA (2008). Fluctuating hemiparesis secondary to moyamoya phenomenon in a child with Down syndrome: a case report. Cases J.

[215] Parker SE, Mai CT, Canfield MA (2010). Updated national birth prevalence estimates for selected birth defects in the United States, 2004-2006. Birth Defects Res A Clin Mol Teratol.

